# Class IIa HDACs inhibit cell death pathways and protect muscle integrity in response to lipotoxicity

**DOI:** 10.1038/s41419-023-06319-5

**Published:** 2023-12-01

**Authors:** Sheree D. Martin, Timothy Connor, Andrew Sanigorski, Kevin A. McEwen, Darren C. Henstridge, Brunda Nijagal, David De Souza, Dedreia L. Tull, Peter J. Meikle, Greg M. Kowalski, Clinton R. Bruce, Paul Gregorevic, Mark A. Febbraio, Fiona M. Collier, Ken R. Walder, Sean L. McGee

**Affiliations:** 1 Institute for Mental and Physical Heath and Clinical Translation (IMPACT) and Metabolic Research Unit, School of Medicine, Deakin University, Geelong, VIC 3216 Australia; 2 College of Health and Medicine, School of Health Sciences, University of Tasmania, Launceston, Australia; 3 Baker Heart and Diabetes Institute, Melbourne, VIC 3004 Australia; 4 Metabolomics Australia, The University of Melbourne, Parkville, VIC 3010 Australia; 5 Institute of Physical Activity and Nutrition (IPAN) and School of Exercise and Nutrition Sciences, Deakin University, Geelong, VIC 3216 Australia; 6 Centre for Muscle Research, Department of Anatomy and Physiology, The University of Melbourne, Parkville, VIC Australia; 7 Monash Institute of Pharmaceutical Sciences, Monash University, Parkville, VIC Australia; 8 Barwon Health, Geelong, Victoria 3220 Australia

**Keywords:** Mechanisms of disease, Obesity

## Abstract

Lipotoxicity, the accumulation of lipids in non-adipose tissues, alters the metabolic transcriptome and mitochondrial metabolism in skeletal muscle. The mechanisms involved remain poorly understood. Here we show that lipotoxicity increased histone deacetylase 4 (HDAC4) and histone deacetylase 5 (HDAC5), which reduced the expression of metabolic genes and oxidative metabolism in skeletal muscle, resulting in increased non-oxidative glucose metabolism. This metabolic reprogramming was also associated with impaired apoptosis and ferroptosis responses, and preserved muscle cell viability in response to lipotoxicity. Mechanistically, increased HDAC4 and 5 decreased acetylation of p53 at K120, a modification required for transcriptional activation of apoptosis. Redox drivers of ferroptosis derived from oxidative metabolism were also reduced. The relevance of this pathway was demonstrated by overexpression of loss-of-function HDAC4 and HDAC5 mutants in skeletal muscle of obese *db/db* mice, which enhanced oxidative metabolic capacity, increased apoptosis and ferroptosis and reduced muscle mass. This study identifies HDAC4 and HDAC5 as repressors of skeletal muscle oxidative metabolism, which is linked to inhibition of cell death pathways and preservation of muscle integrity in response to lipotoxicity.

## Introduction

Obesity is driving an epidemic of chronic disease, including type 2 diabetes. Linked to these diseases are adverse cellular responses arising from the accumulation of lipids in non-adipose tissues, a process termed lipotoxicity [1]. The deleterious effects of lipotoxicity include altered cellular signalling, metabolic reprogramming and cell death [2].

Lipotoxicity alters skeletal muscle metabolism, inducing insulin resistance and impairments in aspects of oxidative metabolism. Reduced rates of both glucose and lipid oxidation have been observed in the skeletal muscle of obese subjects [3, 4] and data describing impairments in amino acid metabolism are also emerging [5–7]. Experimental induction of acute lipotoxicity in humans through lipid infusion results in many of the same skeletal muscle metabolic alterations [8, 9]. These impairments in oxidative metabolism are associated with reduced expression of a metabolic and mitochondrial transcriptional programme controlled by the peroxisome proliferator-activated receptor-gamma coactivator 1 alpha (PGC-1α) transcriptional coactivator [10], encoded by the *PPARGC1A gene*. It is not known why repression of the metabolic transcriptome and reduced oxidative metabolism is an adaptation to lipotoxicity and the molecular pathways involved remain unclear.

Studies from our group and others have found that the class IIa histone deacetylase (HDAC) isoforms 4 and 5 are regulators of PGC-1α expression in skeletal muscle [11, 12]. HDAC4 and 5 act co-operatively as heterodimers to repress the myocyte-enhancer factor 2 (MEF2) family of transcription factors [13, 14]. Binding sites for the MEF2 transcription factor are found in the *PPARGC1A* promoter [12] and in the promoters and enhancer regions of other genes involved in oxidative metabolism [11]. However, the role of the class IIa HDAC transcriptional repressors in the context of lipotoxicity is unknown. We hypothesised that HDAC4 and 5 are increased in response to lipotoxicity and contribute to the suppression of metabolic genes and oxidative metabolism and sought to understand why these adaptations occur.

## Materials and Methods

### Cell culture

Mouse C2C12 myoblasts and myotubes were cultured using standard methods. Cells stably overexpressing HDAC4 and 5 were analysed as myoblasts as HDAC4 and 5 prevented myogenic differentiation of these cells to myotubes. Treatment doses of palmitate, camptothecin, Erastin, 1 S,3R-RSL 3 (Merck, Melbourne, Australia) and Ferrostatin (Selleckchem, Houston, Tx, USA) are reported in Figure legends and were applied for 24 h unless otherwise stated. Palmitate was dissolved in ethanol at 70 °C and diluted in ultrapure H_2_O before conjugation to fatty acid-free BSA at 1:10 ratio (w/v). An ethanol/BSA solution without palmitate was prepared at the same time for use as a vehicle.

### Animal models

All animal experimentation was approved by the Deakin University Animal Ethics Committee (G27-2012 and G21-2015). Male mice were acquired from the Animal Resources Centre (Perth, Australia) and were group housed with a 12 hr light/dark cycle, temperature 21 ± 3 °C, humidity 30–70% with ad libitum access to standard rodent chow and water. Heterozygous *db/+* and homozygous *db/db* mice on a C57BL/6 J background were fasted for 5 h prior to humane sacrifice by cervical dislocation for rapid excision of tissues, which were snap frozen in liquid nitrogen. C57BL/6J mice were administered recombinant serotype 6 adeno-associated viral (AAV6) (rAAV6) vectors via intramuscular injection into the anterior and posterior compartments of the lower hind limbs. Each compartment of the left limb received 2e^10^ vector genomes (vg) of empty rAAV6, whilst each compartment of the right limb received 1e^10^ vg of WT HDAC4 rAAV6 and 1e^10^ vg of WT HDAC5 rAAV6. *db/db* mice were similarly administered rAAV6 vectors via intramuscular injection. Each compartment of the left limb received 5e^10^ vg of empty rAAV6, whilst each compartment of the right limb received 2.5e^10^ vg of DN HDAC4 rAAV6 and 2.5e^10^ vg of DN HDAC5 rAAV6. Experiments were performed ~8 weeks after rAAV6 administration. At the conclusion of experiments mice were fasted for 4 hours before humane killing by cervical dislocation. Tissues were rapidly excised and snap frozen in liquid nitrogen or fixed in 10% formalin.

### Respiration and mitochondrial function analysis

Mitochondrial function in cells treated with vehicle (BSA) or palmitate was assessed as previously described [15] and respiratory analysis of skeletal muscle biopsies was also performed as previously described [11].

### Lipidomics and metabolite analysis

To determine cellular lipid profiles, ~1e^6^ cells were collected in 100 µl of PBS and frozen at −80 °C, or ~10 mg of muscle was homogenised in 100 µl of PBS, and lipids were determined as previously described [16]. Briefly, lipids were extracted using 20 volumes of chloroform:methanol (2:1) in a single-phase extraction process, recovering all lipids in a single phase suitable for liquid chromatography–mass spectrometry analysis. Lipids were identified using a HP 1200 liquid chromatography system (Agilent Technologies) coupled to a PE Sciex API 4000 Q/TRAP mass spectrometer (Applied Biosystems/MDS SCIEX, Mulgrave, VIC, Australia) with a turbo-ionspray source (350 °C) and Analyst 1.5 data system (Applied Biosystems/MDS SCIEX). Quantification of individual lipid species was performed using multiple-reaction monitoring (MRM) in positive ion mode. MRM product ions used were m/z 264 for ceramides, while diacylglycerides and triacylglycerides were monitored by the neutral loss of individual fatty acid species. Each ion pair was monitored for between 10 and 50 ms (using scheduled MRM mode), with a resolution of 0.7 amu at half-peak height, and averaged from continuous scans over the elution period. The proportionately higher signals resulting from diacylglycerol and triacylglycerol consisting of two or more identical fatty acids were corrected prior to normalisation against internal standards. Lipid concentrations were calculated by relating the peak area of each species to the peak area of the corresponding internal standards (100 pmol ceramide 17:0, 200 pmol diacylglycerol 17:0/17:0 and 100 pmol triacylglycerol 17:0/17:0/17:0), which were added prior to extraction. For detection of Glu and GSH, cells in six-well plates underwent metabolic arrest by washing in 1 mL ultrapure water before being frozen instantaneously by the addition of liquid nitrogen. To extract polar metabolites, ice-cold methanol:chloroform (9:1) containing internal standards (0.5 nM 13C-sorbitol and 5 nM 13 C, 15N-valine) was added. Metabolites were detected using LC and high-resolution QTOF mass spectrometry previously described [17] with the following modifications. Samples (10 μL) were injected onto an Agilent 1290 LC fitted with a SeQuant ZIC®-pHILIC column (2.1 × 150 mm, 5μm; Merck) using 20 mM ammonium carbonate, pH 9.0 (Sigma-Aldrich) and 100% acetonitrile as the mobile phases.

### Microarray and gene expression analysis

For comparative transcriptomics analysis, each group consisted of three biological replicates and microarray analysis was performed as previously described [11] and conform to MIAME guidelines. Genes found to be significantly altered in the same direction in both cell and skeletal muscle data sets (*n* = 236) were analysed by gene set enrichment analysis using KEGG pathways. Gene expression was measured using real time RT-PCR as previously described [18]. Primer sequences are available in Table S1.

### Immunoprecipitation and western blot analysis

Cells were collected in lysis buffer (50 mM Tris pH 7.5, 1 mM EDTA, 1 mM EGTA, 10% glycerol, 1% Triton X-100, 50 mM NaF, 5 mM sodium pyrophosphate, 1 mM DTT, 1X protease inhibitor cocktail (Merck)) and ~20 mg skeletal muscle was homogenised in 10 volumes of lysis buffer using a handheld homogeniser. Protein concentration was determined using the BCA assay (Pierce). Immunoprecipitation and western blotting were performed as previously described [19]. Antibody details are listed in Table S2.

### Insulin/isotopic glucose administration and stable isotope metabolomics

Mice were administered 200 μCi/kg of 2[1,2-^3^H]-deoxyglucose (2-DG) and 100 μCi/kg of 1-^14^C glucose, with or without 1.2 U/kg insulin, following a 5 h fast. Blood glucose was determined using a handheld glucometer (Accu-Chek Performa, Merck) and ~5 μL blood was obtained from the tail tip prior to tracer administration and 5, 15 and 30 min after tracer administration. Plasma tracer concentration and tissue 2-deoxyglucose clearance were determined as we have previously described [20]. To determine 1-^14^C glucose incorporation into glycogen, ~10–15 mg of tibialis anterior muscle was digested in 1 M KOH at 70 °C for 20 min and glycogen was precipitated with saturated Na_2_SO_4_, washed twice with 95% ethanol and resuspended in acetate buffer (0.84% sodium acetate, 0.46% acetic acid, pH 4.75) containing 0.3 mg/mL amyloglucosidase (Merck). Glycogen was digested overnight at 37 °C before glucose content was quantified using the glucose oxidase method [15] and ^14^C-glucose incorporation was measured. To determine 1-^14^C glucose incorporation into lipids, 5–10 mg of tibialis anterior (TA) muscle was homogenised in chloroform/methanol (2:1) and mixed overnight at room temperature. Organic and inorganic phases were separated, and the lower organic phase was collected and evaporated under N_2_ at 45 °C. The dried extract was resuspended in absolute ethanol and TG content was assayed using TG GPO-PAP reagent (Merck) and ^14^C-glucose incorporation was measured.

To assess glucose utilisation by metabolic pathways, mice were administered a bolus of 50 mg of [U-^13^C] glucose (Sigma-Aldrich) via oral gavage. At 60 min later, mice were humanely killed by cervical dislocation and skeletal muscles were immediately collected. Targeted metabolomics and analysis of [U-^13^C] labelling was performed as we have previously described [20].

### Measurement of apoptosis and cell viability

Analysis of apoptotic cells was performed by dual staining with FITC-conjugated Annexin V (Merck) and propidium iodide (PI; Thermo-Fischer, Waltham, MA, USA) and was optimised for adherent cells. For measurement of apoptosis in tissues, caspase 3 activity was measured using Caspase-Glo 3/7 assay (Promega, Alexandria, Australia). Quantification of viable cells was performed by staining with 7-aminoactinomycin D (7-AAD; Merck) and flow cytometry or with crystal violet staining with colorimetric detection.

### Reactive oxygen species detection

In cells, hydrogen peroxide (H_2_O_2_) was detected using Amplex Red assay (Invitrogen, Mt Waverly, Australia). Cells were seeded into a 96-well plate at 25,000 cells/well. The following day, cells were treated with vehicle (DMSO), 100 µM mitoTEMPO or 100 µM apocynin for 30 min at 37 °C. Cells were then co-incubated with 50 µM Amplex Red and 0.1 U/ml horseradish peroxidase (HRP) for a further 30 min at 37 °C. Mitochondrial ROS was designated as that sensitive to MitoTEMPO, while NADPH oxidase ROS was designated as that sensitive to apocynin. For tissues, the OxiSelect In Vitro ROS/RNS Assay kit (Green Fluorescence) (Cell Biolabs Inc., STA-347) was used according to the manufacturer’s instructions.

### GPX4 activity and lipid peroxidation

Glutathione-dependent peroxidase activity was measured using a colorimetric Glutathione Peroxidase Assay Kit (AbCam, ab102530). Cells were plated into 10 cm tissue culture plates at 4 × 10^6^ cells per dish. After 3 h, media was refreshed and cells incubated for 1 h, washed and collected in 250 μl of supplied assay buffer. For tissues, muscle lysate was analysed. Data was then analysed as described by the manufacturer with results normalised to protein concentration. Lipid peroxidation in both cells and muscle lysates was assessed using a lipid peroxidation kit (Sigma, MAK-085). The assay was performed according to manufacturer’s instructions, including addition of butanol to the reaction.

### Histology

Fixed TA muscles were embedded in paraffin before transverse sections were obtained at 5 μm thickness and stained for H&E and Masson’s trichrome. Slides were scanned (Aperio, Leica Biosystems) and were analysed using photoshop and image J.

### Statistical analysis

Individual data points represent biological replicates for both animal (individual mouse) and cell (separate culture dish/well) experiments. All data are expressed as mean ± SEM. Individual data points identified as greater than two standard deviations away from the mean were designated as outliers and removed. Data was assessed for normality using the Shapiro-Wilk test. Differences between groups were assessed using *t*-test, Kruskal-Wallis test, one-way ANOVA or two-way ANOVA as appropriate using GraphPad Prism. Specific differences between groups were identified using Dunn’s or Tukey’s multiple comparisons tests. For animal experiments where opposing hind limbs were administered different rAAVs, such that control and experimental conditions were contained within the one animal, paired *t*-tests were used. Differences were considered statistically significant where *p* < 0.05.

## Results

### HDAC4 and 5 are increased in models of lipotoxicity

**Fig. 1:**
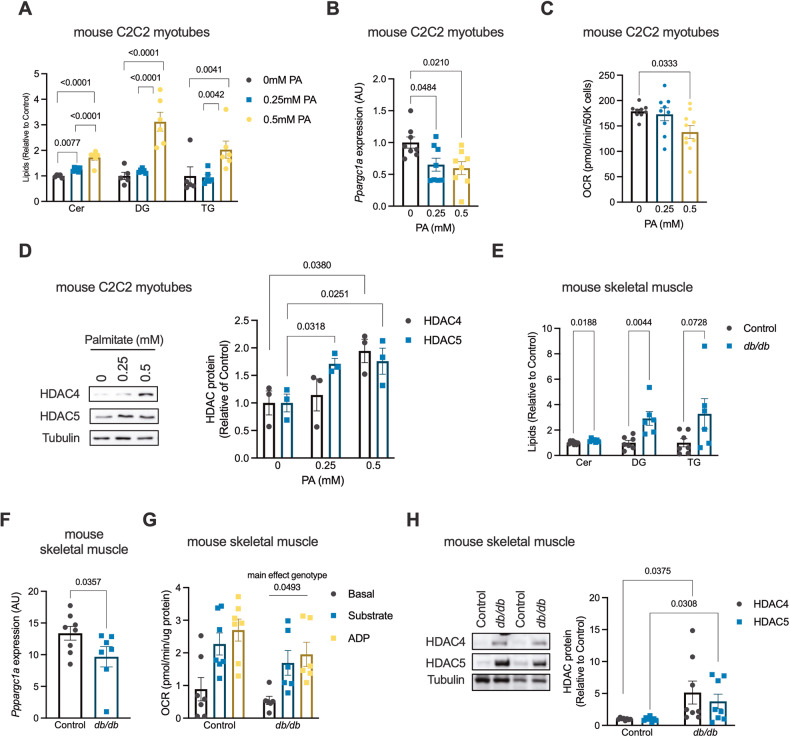
HDAC4 and 5 are increased in models of lipotoxicity. **A** Total Ceramide, Diglyceride (DG) and triglyceride (TG) lipids in C2C12 myotubes treated with 0 mM (BSA vehicle), 0.25 mM or 0.5 mM palmitate (PA) for 16 h (One-way ANOVA; Cer *p* < 0.0001 (*F*(2,14) = 73.86), DG *p* < 0.0001 (*F*(2,14) = 38.50), TG *p* < 0.0017 (*F*(2,14) = 10.38), significant Tukey’s multiple comparisons shown). **B** *Ppargc1a* gene expression in C2C12 myotubes treated with 0 mM, 0.25 mM or 0.5 mM PA for 16 h (One-way ANOVA *p* < 0.0161 (*F*(2,21) = 5.054), significant Tukey’s multiple comparisons shown). **C** Oxygen consumption rate (OCR) in C2C12 myotubes treated with 0 mM, 0.25 mM or 0.5 mM PA for 16 h (One-way ANOVA *p* < 0.0255 (*F*(2,25) = 4.262), significant Tukey’s multiple comparisons shown). **D** Representative western blots and HDAC4 and 5 protein in C2C12 myotubes treated with 0 mM, 0.25 mM or 0.5 mM PA for 16 h (HDAC4, Kruskal-Wallis test, *p* = 0.049 (*X*^2^ = 5.591), significant Dunn’s multiple comparison shown; HDAC5, One-way ANOVA, *p* = 0.038 (*F*(2,6) = 5.903), significant Tukey’s multiple com*p*arisons shown). **E** Total Cer, DG and TG lipids in tibialis anterior (TA) skeletal muscle of Control and *db/db* mice (Unpaired *t*-tests). **F** *Ppargc1a* gene expression in TA skeletal muscle of Control and *db/db* mice. **G** Basal, substrate and ADP-driven OCR in biopsies of the gastrocnemius skeletal muscle of Control and *db/db* mice (Two-way ANOVA, genotype *F*(1,33) = 4.166). **H** Representative western blots (two biological replicates per group) and HDAC4 and 5 protein in TA skeletal muscle of Control and *db/db* mice (Unpaired *t*-tests). Data are mean ± SEM, *n* = 3–6 biological replicates per group for cell experiments and 6–8 per group for animal experiments.

To test the hypothesis that HDAC4 and 5 are increased by lipotoxicity, C2C12 myotubes were exposed to 0.25 or 0.5 mM palmitate, or BSA (vehicle), for 16 h. Application of 0.5 mM palmitate-induced alterations consistent with lipotoxicity in muscle cells, including increased ceramide, diglyceride (DG) and triglyceride (TG) levels (Figs. 1A and S1A), lower *Ppargc1a* gene expression (Fig. 1B) and a reduction in cellular respiration (Fig. 1C). The abundance of both HDAC4 and HDAC5 protein was increased by 0.5 mM palmitate, while HDAC5 protein abundance was also increased by 0.25 mM palmitate (Fig. 1D). There were no differences in *Hdac4* or *Hdac5* gene expression between groups (Fig. S1B).

To examine whether skeletal muscle HDAC4 and 5 are increased in an in vivo model of lipotoxicity, the TA skeletal muscle of obese *db/db* and control heterozygous littermate mice was analysed. Total ceramide and DG were increased in skeletal muscle of *db/db* mice, while there was a trend for TG to also be higher (Figs. 1E and S1C). Increased muscle lipid concentrations were associated with decreased *Ppargc-1α* gene expression (Fig. 1F), a main effect for reduced skeletal muscle respiratory responses (Fig. 1G) and increased HDAC4 and 5 protein abundance (Fig. 1H). Skeletal muscle gene expression levels of *Hdac5*, but not *Hdac4*, were increased in *db/db* mice (Fig. S1D). Notwithstanding the complex physiology of the *db/db* model, these data suggest that skeletal muscle HDAC4 and 5 are increased in response to lipotoxicity and are associated with reduced *Ppargc1a* expression and impaired oxidative metabolism.

### Overexpression of HDAC4 and 5 in skeletal muscle represses *Pppargc1a* expression and oxidative capacity

**Fig. 2:**
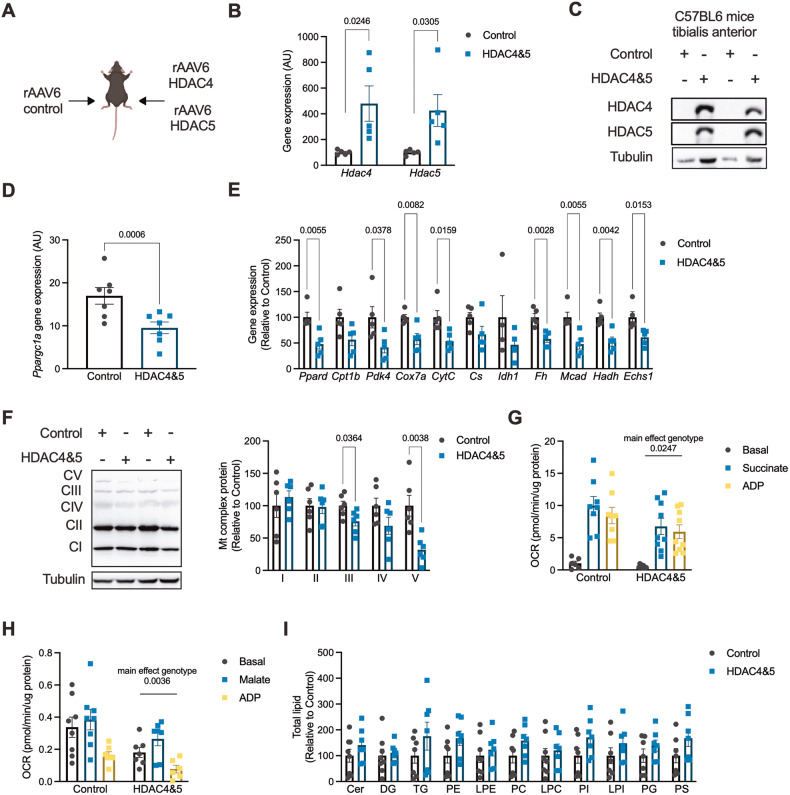
Overexpression of HDAC4 and 5 in skeletal muscle suppresses *Pppargc1a* expression and oxidative capacity. **A** The bilateral AAV6 HDAC4 and 5 overexpression mouse model. **B** *Hdac4* and *Hdac5* gene expression in Control and HDAC4 and 5 overexpressing tibialis anterior (TA) skeletal muscle (Paired *t*-tests). **C** HDAC4 and 5 protein in Control and HDAC4 and 5 overexpressing TA skeletal muscle. **D** *Ppargc1a* gene expression in Control and HDAC4 and 5 overexpressing TA skeletal muscle (Paired *t*-tests). **E** Metabolic gene expression in Control and HDAC4 and 5 overexpressing TA skeletal muscle (Paired *t*-tests). **F** Respiratory chain complex subunit protein in Control and HDAC4 and 5 overexpressing TA skeletal muscle (Paired *t*-tests). **G** Basal, succinate and ADP-driven oxygen consumption rate (OCR) from biopsies of Control and HDAC4 and 5 overexpressing gastrocnemius skeletal muscle (Two-way ANOVA, genotype *F*(1,43) = 5.417). **H** Basal, malate and ADP-driven OCR from biopsies of Control and HDAC4 and 5 overexpressing gastrocnemius skeletal muscle (Two-way ANOVA, genotype *F*(1,37) = 9.661). **I** Ceramide (Cer), diglyceride (DG), triglyceride (TG), phosphatidylethanolamine (PE), lysophosphatidylethanolamine (LPE), phosphatidylcholine (PC), lysophosphatidylcholine (LPC), phosphatidylinositol (PI), lysophosphatidylinositol (LPI), phosphoglycerol (PG) and phosphatidylserine (PS) lipids in Control and HDAC4 and 5 overexpressing TA skeletal muscle. Data are mean ± SEM, *n* = 4–6 per group.

To examine whether increased HDAC4 and 5 impacts skeletal muscle metabolism, a bilateral skeletal muscle HDAC4 and 5 mouse model was developed using rAAV6 vectors expressing HDAC4 and HDAC5 (Fig. 2A). In TA muscle, this model had increased *Hdac4* and *Hdac5* gene expression (Fig. 2B) and protein (Fig. 2C). Overexpression of HDAC4 and 5 reduced *Ppargc1a* expression (Fig. 2D) and the expression of a range of MEF2 and PGC-1α target genes involved in oxidative and mitochondrial metabolism (Fig. 2E). These changes in metabolic transcripts were associated with reduced protein abundance of subunits of complex III and V (ATP synthase), but not other subunits of electron transport chain complexes (Fig. 2F). Reduced ATP synthase components was associated with main effects for reduced respiratory responses in muscles overexpressing HDAC4 and 5 when driven by both succinate (Fig. 2G) and malate (Fig. 2H) as substrates. Overexpression of HDAC4 and 5 was not associated with statistically significant accumulation of lipids (Fig. 2I), although lipid levels were generally higher with overexpression of HDAC4 and 5. These data indicate that increasing HDAC4 and 5 represses *Ppargc1a* gene expression and oxidative capacity in skeletal muscle.

### Overexpression of HDAC4 and 5 in skeletal muscle inhibits oxidative glucose utilisation

**Fig. 3:**
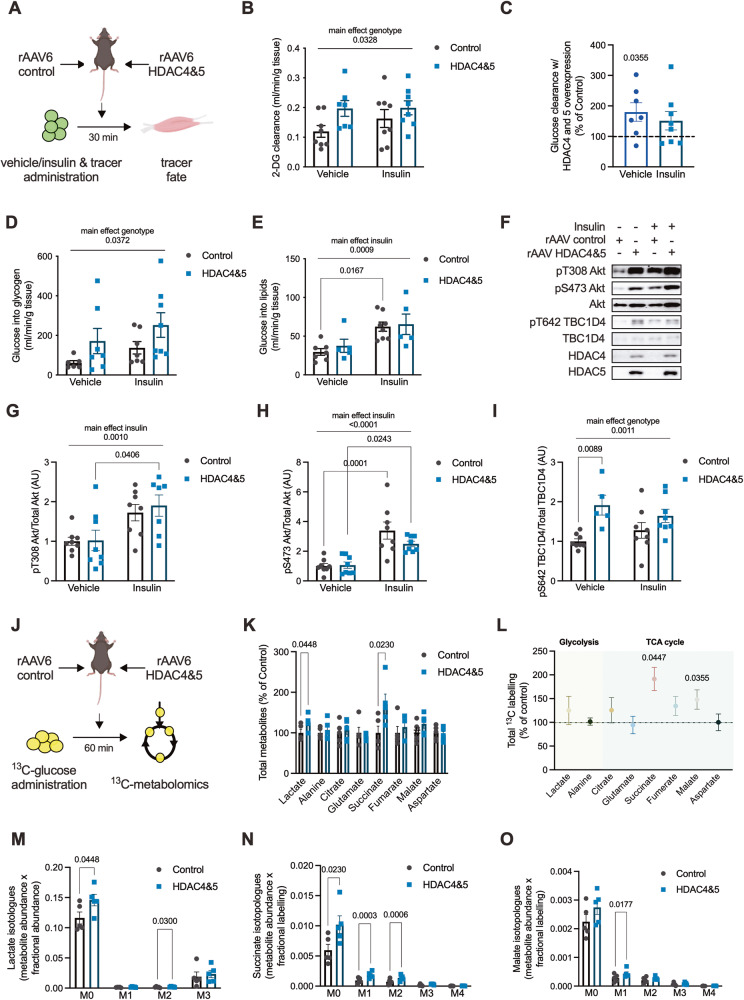
Overexpression of HDAC4 and 5 in skeletal muscle inhibits oxidative glucose utilisation. **A** Experimental overview of vehicle/insulin and isotopic glucose tracer administration in the bilateral AAV6 HDAC4 and 5 overexpression model. **B** 2-deoxyglucose (2-DG) clearance in tibialis anterior (TA) skeletal muscle of bilateral AAV6 HDAC4 and 5 mice administered vehicle or insulin and isotopic glucose tracers (Two-way ANOVA, genotype *F*(1,27) = 5.061). **C** 2-DG clearance in TA skeletal muscle overexpressing HDAC4 and 5 relative to Control (Paired *t*-test). **D** ^14^C-glucose incorporation into glycogen in TA skeletal muscle of bilateral AAV6 HDAC4 and 5 mice administered vehicle or insulin and isotopic glucose tracers (Two-way ANOVA, genotype *F*(1,24) = 4.864). **E** ^14^C-glucose incorporation into lipid in TA skeletal muscle of bilateral AAV6 HDAC4 and 5 mice administered vehicle or insulin and isotopic glucose tracers (Two-way ANOVA, insulin *F*(1,21) = 14.94). **F** Representative images of insulin signalling components in TA skeletal muscle of bilateral AAV6 HDAC4 and 5 mice administered vehicle or insulin and isotopic glucose tracers and quantification of (**G**) pT308 Akt (Two-way ANOVA, insulin *F*(1,27) = 13.75); (**H**) pS473 Akt (Two-way ANOVA, insulin *F*(1,28) = 33.13), and; (**I**) pS642 TBC1D4 (Two-way ANOVA, genotype *F*(1,25) = 13.60). **J** Experimental overview of stable isotope metabolomics in the bilateral AAV6 HDAC4 and 5 overexpression model administered U^13^C-glucose. **K** Total metabolite abundance in TA skeletal muscle of bilateral AAV6 HDAC4 and 5 mice administered U^13^C-glucose. (Paired *t*-tests). **L** Total ^13^C labelling of glycolytic and TCA cycle metabolites in TA skeletal muscle of bilateral AAV6 HDAC4 and 5 mice administered U^13^C-glucose. (Paired *t*-tests). Isotopologues of (**M**) Lactate; (**N**) Succinate, and; (**O**) Malate (Paired *t*-tests) in TA skeletal muscle of bilateral AAV6 HDAC4 and five mice administered U^13^C-glucose. Data are mean ± SEM, *n* = 5–8 per group.

Reduced skeletal muscle oxidative capacity associated with lipotoxicity is linked with reduced insulin action and impaired glucose utilisation [4, 21, 22]. To determine whether HDAC4 and 5 cause insulin resistance, bilateral skeletal muscle HDAC4 and 5 mice were administered either vehicle or insulin and glucose tracers (Fig. 3A). Insulin reduced the excursion of both glucose tracers (Fig. S2A, B). There was a main effect for increased glucose clearance in skeletal muscles overexpressing HDAC4 and 5 (Fig. 3B). When expressed relative to control skeletal muscle within each mouse, skeletal muscle with HDAC4 and 5 overexpression had increased glucose clearance in vehicle but not insulin-stimulated conditions (Fig. 3C). Examination of glucose fate revealed a main effect for overexpression of HDAC4 and 5 to increase glucose incorporation into glycogen (Fig. 3D) and increase glycogen concentration (Fig. S2C). In contrast, there was a main effect for insulin to increase glucose incorporation into lipids (Fig. 3E), which was associated with a main effect for insulin to increase Akt phosphorylation at T308 (Fig. 3F, G) and S473 (Fig. 3F, H). Consistent with the increase in glucose clearance, phosphorylation of TBC1D4 at S642, which is required for GLUT4 translocation to the plasma membrane [23], was increased in skeletal muscle overexpressing HDAC4 and 5 (main effect; Fig. 3I). These data indicate that increases in skeletal muscle HDAC4 and 5 increase glucose utilisation through non-oxidative pathways such as glycogen synthesis.

To determine whether HDAC4 and 5 inhibits glucose utilisation through oxidative pathways, a targeted stable isotope metabolomics approach in bilateral skeletal muscle HDAC4 and 5 mice was employed (Fig. 3J). Overexpression of HDAC4 and 5 increased the total abundance of lactate and succinate (Fig. 3K) and total ^13^C labelling of succinate and malate (Fig. 3L). Isotopologue analysis of lactate, succinate and malate was performed to provide further insight into the metabolic reprogramming induced by HDAC4 and 5 overexpression. This revealed accumulation of both M + 0 and M + 2 lactate in muscle overexpressing HDAC4 and 5 (Fig. 3M), suggesting increased non-oxidative glucose utilisation and increased malate/pyruvate cycle flux, respectively, which can be indicative of defective TCA cycle function [24]. Succinate M + 0, M + 1 and M + 2 isotopologues were increased in muscle overexpressing HDAC4 and 5 (Fig. 3N), consistent with a major impairment in succinate dehydrogenase (SDH) activity [25], which is bifunctional for both the TCA cycle and the electron transport chain. These data are also supported by reduced succinate-driven respiration in muscles overexpressing HDAC4 and 5 (Fig. 2G). Similarly, HDAC4 and 5 overexpression was associated with increased malate M + 0 and M + 1 isotopologues (Fig. 3O). Together, the data from both tracer studies indicate that increased HDAC4 and 5 is linked with defective oxidative glucose utilisation and increased non-oxidative glucose utilisation in skeletal muscle.

### HDAC4 and 5 enhance cell survival in response to lipotoxicity and supress the apoptosis and ferroptosis cell death pathways

To better understand why this metabolic reprogramming occurs, important genes regulated by HDAC4 and 5 in the context of lipotoxicity were identified using a comparative transcriptomics approach. Specifically, the transcriptome altered in the skeletal muscle of *db/db* mice in which HDAC4 and 5 was increased (Fig. 1H) was compared to the transcriptome altered in myogenic C2C12 myoblasts stably overexpressing HDAC4 and 5 (Fig. S3A). Overexpression of HDAC4 and 5 in these cells reduced *Ppargc1a* gene expression, suppressed OXPHOS and TCA cycle genes, and reduced oxidative capacity (Fig. S3B–D). The comparative transcriptomic analysis and subsequent pathway analysis of coregulated genes revealed the p53 signalling pathway related to cell survival as the only significantly regulated pathway (Fig. S3E).

**Fig. 4:**
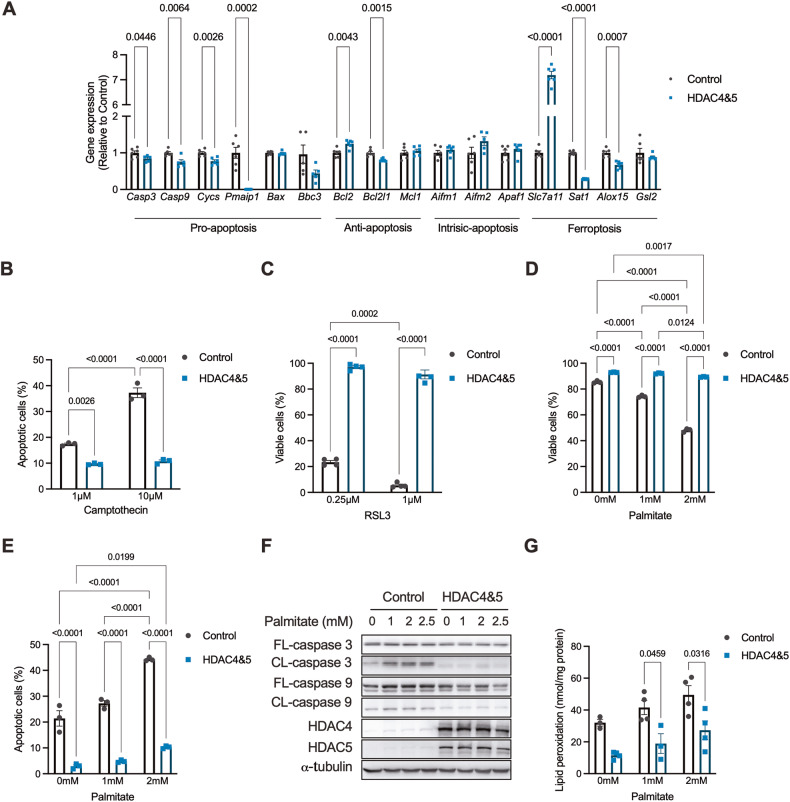
HDAC and 5 enhance cell survival in response to lipotoxicity and supress the apoptosis and ferroptosis cell death pathways. **A** Expression of p53-pathway genes involved in pro-apoptosis, anti-apoptosis, intrinsic apoptosis and ferroptosis in Control and HDAC4 and 5 overexpressing C2C12 myoblasts. (Unpaired *t*-tests). **B** Apoptotic cells in response to the apoptosis-inducing agent Camptothecin in Control and HDAC4 and 5 overexpressing C2C12 myoblasts. (Two-way ANOVA, genotype *p* < 0.0001 *F*(1,12) = 349.3, treatment *p* < 0.0001 *F*(2,12) = 270.4, interaction *p* < 0.0001 *F*(2,12) = 149.5, significant Tukey’s multiple comparisons shown). **C** Cell viability in response to the ferroptosis-inducing agent RSL3 in Control and HDAC4 and 5 overexpressing C2C12 myoblasts. (Two-way ANOVA, genotype *p* < 0.0001 *F*(1,12) = 1645, treatment *p* < 0.0001 *F*(1,12) = 36.09, interaction *p* < 0.0090 *F*(1,12) = 9.665, significant Tukey’s multiple com*p*arisons shown). **D** Cell viability in response to palmitate in Control and HDAC4 and 5 overexpressing C2C12 myoblasts (Two-way ANOVA, genotype *p* < 0.0001 *F*(1,12) = 3374, treatment *p* < 0.0001 *F*(2,12) = 1012, interaction *p* < 0.0001 *F*(2,12) = 684.8, significant Tukey’s multiple comparisons shown). **E** Apoptotic cells in response to palmitate in Control and HDAC4 and 5 overexpressing C2C12 myoblasts. (Two-way ANOVA, genotype *p* < 0.0001 *F*(1,12) = 1468, treatment *p* = 0.0001 *F*(2,12) = 20.35, interaction *p* < 0.0050 *F*(2,12) = 8.498, significant Tukey’s multiple comparisons shown). **F** Western blots of full-length (FL) and cleaved (CL) caspase 3 and 9 in response to increasing palmitate concentrations in Control and HDAC4 and 5 overexpressing C2C12 myoblasts. **G** Lipid peroxidation in response to palmitate (Two-way ANOVA, genotype *p* < 0.0001 *F*(1,12) = 313.4, treatment *p* < 0.0103 *F*(2,12) = 67.31, significant Tukey’s multiple comparisons shown) in Control and HDAC4 and 5 overexpressing C2C12 myoblasts. Data are mean ± SEM, *n* = 3–4 biological replicates per group.

Profiling of downstream genes in this pathway in C2C12 myoblasts overexpressing HDAC4 and 5 revealed that several pro-apoptosis genes were reduced, including *Casp3* and *9, Cycs, Pmaip1* and *Bbc3* (Fig. 4A), consistent with suppression of apoptosis. Similarly, genes involved in ferroptosis, a form of iron-mediated cell death involving lipid peroxidation [26], were altered in ways that indicated suppression of ferroptosis, including increased *Slc7a11* expression and reduced *Sat1* and *Alox15* expression (Fig. 4A). These data suggest that increased HDAC4 and 5 inhibit apoptotic and ferroptotic cell death pathways. Indeed, overexpression of HDAC4 and 5 in C2C12 myoblasts reduced the number of apoptotic cells following exposure to the apoptosis-inducing agent camptothecin (Fig. 4B), as assessed by Annexin V/PI staining. Similarly, cell viability was higher in cells overexpressing HDAC4 and 5 following exposure to the ferroptosis-inducing agent RSL3 (Fig. 4C). These data raised the possibility that suppression of the apoptosis and ferroptosis pathways by HDAC4 and 5 enhances cell survival in response to lipotoxicity. To test this hypothesis, cells were exposed to increasing concentrations of palmitate intended to challenge cell viability. Overexpression of HDAC4 and 5 enhanced the viability of cells exposed to both 1 and 2 mM of palmitate compared with control cells and resulted in cells being largely resistant to lipotoxicity (Fig. 4D). This protection of cell viability was associated with fewer apoptotic cells in response to increasing concentrations of palmitate (Fig. 4E), while cleavage of caspase 3 and 9, the irreversible events that trigger apoptosis, was nearly undetectable by western blot in these cells under the same conditions (Fig. 4F). Overexposure of western blots showed that endogenous HDAC4 and 5 were increased by these high concentrations of palmitate (Fig. S3F), similar to the lower concentrations of palmitate characterised previously (Fig. 1D). Similarly, overexpression of HDAC4 and 5 reduced lipid peroxidation, the trigger for ferroptosis induced-cell death, in response to increasing concentrations of palmitate (Fig. 4G). These data indicate that increased HDAC4 and 5 inhibit the apoptosis and ferroptosis cell death pathways and enhance cell viability in response to lipotoxicity.

### HDAC4 and 5 inhibit apoptosis by reducing p53 K120 acetylation

**Fig. 5:**
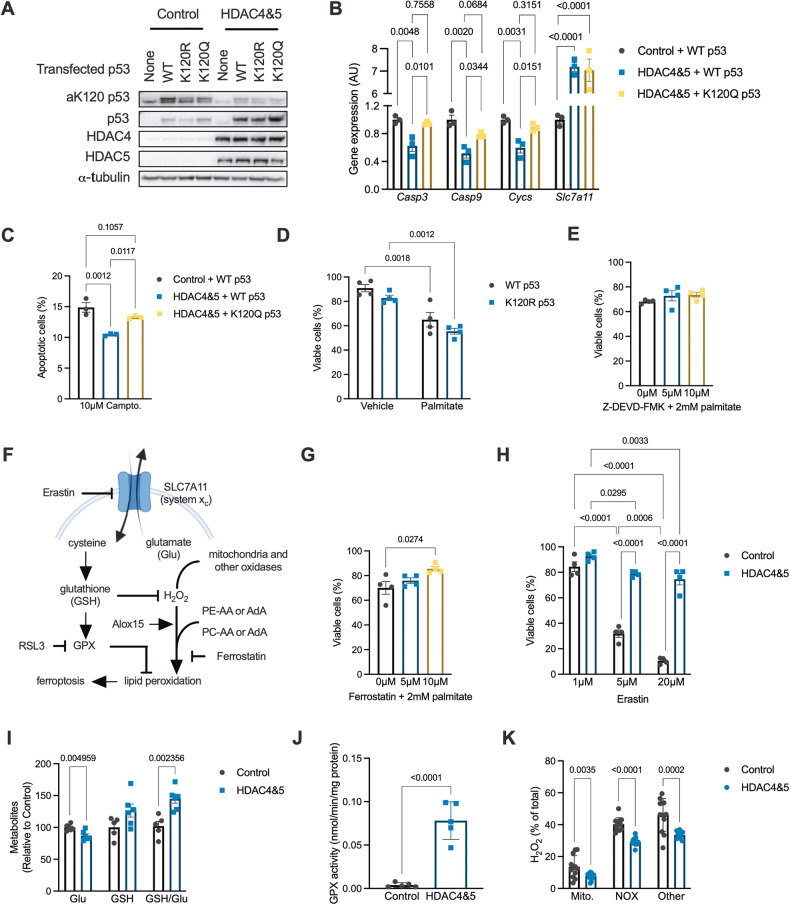
HDAC4 and 5 inhibit apoptosis by reducing p53 K120 acetylation and reduces multiple metabolic inputs to the ferroptosis pathway. **A** Acetylated p53 at lysine 120 (aK120 p53) in Control or HDAC4 and 5 overexpressing C2C12 myoblasts also expressing wild type (WT), K120R (loss-of-function) or K120Q (gain-of-function) p53. **B** Expression of caspase 3 (*Casp3;* One-way ANOVA *p* = 0.0040 *×*^*2*^ = 0.8408), caspase 9 (*Casp9;* One-way ANOVA *p* = 0.0025 *×*^*2*^ = 0.8650), cytochrome c (*Cycs;* One-way ANOVA *p* = 0.0033 *×*^*2*^ = 0.8518) and the cystine/glutamate antiporter xCT (*Slc7a11;* One-way ANOVA *p* < 0.0001 *×*^*2*^ = 0.9769) in Control or HDAC4 and 5 overexpressing C2C12 myoblasts also expressing WT or K120Q p53 (Tukey’s multiple comparisons shown). **C** Percentage of apoptotic cells following exposure to camptothecin in Control or HDAC4 and 5 overexpressing C2C12 myoblasts also expressing WT or K120Q p53 (One-way ANOVA *p* = 0.0014 *×*^*2*^ = 0.8875, Tukey’s multiple comparisons shown). **D** Cell viability 16 h after palmitate exposure in C2C12 myoblasts expressing WT or K120Q p53 (Two-way ANOVA, treatment *p* < 0.0001 *F*(1,12) = 50.44, genotype *p* = 0.0361 *F*(1,12) = 5.56, significant Tukey’s multiple comparisons shown). **E** Cell viability in C2C12 myoblasts following palmitate exposure and co-incubation with vehicle (DMSO) or increasing concentrations of Z-DEVD-FMK (One-way ANOVA, *p* = 0.3282). **F** Schematic showing key regulatory points in the ferroptosis pathway. **G** Cell viability in C2C12 myoblasts following palmitate exposure and co-incubation with vehicle (DMSO) or increasing concentrations of Ferrostatin (One-way ANOVA, *p* = 0.0323 *×*^*2*^ = 0.5337, significant Tukey’s multiple comparisons shown). **H** Cell viability in Control or HDAC4 and 5 overexpressing C2C12 myoblasts following exposure to increasing concentrations of Erastin (Two-way ANOVA, genotype *p* < 0.0001 *F*(1,18) = 293.7, treatment *p* < 0.0001 *F*(2,18) = 139.6, interaction *p* < 0.0001 *F*(2,18) = 50.88, significant Tukey’s multiple comparisons shown). **I** Glutamate (Glu), glutathione (GSH) and the GSH/Glu ratio in Control or HDAC4 and 5 overexpressing C2C12 myoblasts (Unpaired *t*-tests). **J** Glutathione peroxidase (GPX) activity in Control or HDAC4 and 5 overexpressing C2C12 myoblasts (Unpaired *t*-test). **K** H_2_O_2_ derived from mitochondria (mito), NADPH oxidases (NOX) and other sources in Control or HDAC4 and 5 overexpressing C2C12 myoblasts (Unpaired *t*-tests). Data are mean ± SEM, *n* = 4–10 biological replicates per group.

In several cancer cell types, HDAC5 has been linked with reduced acetylation of p53 at K120, which inhibits p53 transcriptional activity and the expression of apoptosis genes, which reduces apoptosis sensitivity [27–29]. Furthermore, acetylation of p53, including at K120, can contribute to the transcriptional regulation of ferroptosis [30]. To explore whether reduced acetylation of p53 at K120 is involved in suppression of apoptosis and ferroptosis by HDAC4 and 5 in myogenic cells, C2C12 myoblasts stably overexpressing HDAC4 and 5 were transfected with wild-type p53, or p53 in which K120 was mutated to either glutamine (K120Q; acetylation mimetic gain-of function) or arginine (K120R; deacetylation mimetic loss-of-function) (Fig. 5A). The acetylation of p53 at K120 was reduced by the overexpression of HDAC4 and 5 (Fig. 5A). In cells overexpressing HDAC4 and 5, introduction of the p53^K120Q^ acetylation mimetic mutant restored the expression of the pro-apoptosis genes *Casp3, Casp9* and *Cycs* (Fig. 5B). However, the expression of *Slc7a11*, which opposes ferroptosis, was not influenced by expression of this mutant (Fig. 5B). This suggests that reduced acetylation of p53 at K120 by HDAC4 and 5 suppresses apoptosis, but not ferroptosis, transcriptional programmes. Expression of p53^K120Q^ also restored apoptosis in HDAC4 and 5 overexpressing cells exposed to the apoptosis-inducing agent camptothecin (Fig. 5C). Although p53 is also known to regulate aspects of cellular metabolism [31], gain and loss-of-function K120 p53 mutants had no effect on either oxidative (Fig. S4A) or glycolytic flux (Fig. S4B). To determine whether p53 K120 acetylation determines cell viability in response to lipotoxicity, cells expressing WT p53 or p53^K120R^ were exposed to 2 mM palmitate. However, the p53^K120R^ mutant did not influence cell viability in response to palmitate exposure (Fig. 5D). Furthermore, the apoptosis inhibitor, Z-DEVD-FMK, had no effect on cell viability in response to palmitate exposure (Fig. 5E), suggesting that other pathways in addition to apoptosis are also involved in the cell death response to lipotoxicity.

### HDAC4 and 5 inhibit multiple metabolic inputs to the ferroptosis pathway

The role of ferroptosis-mediated mechanisms of cell death (Fig. 5F) was further explored. The ferroptosis inhibitor Ferrostatin enhanced C2C12 myoblast viability following exposure to 2 mM palmitate (Fig. 5G), indicating an important role for ferroptosis-mediated cell death in response to lipotoxicity. Overexpression of HDAC4 and 5 enhanced cell viability in response to increasing concentrations of Erastin (Fig. 5H), an agent that induces ferroptosis by inhibiting system x_c_ and glutamate and cysteine exchange [32], which in turn reduces glutathione (GSH) levels and results in lipid peroxidation through multiple mechanisms (Fig. 5F). Consistent with HDAC4 and 5 increasing the expression of *Slc7a11* (Fig. 4A), overexpression of HDAC4 and 5 reduced intracellular glutamate concentrations, tended to increase intracellular GSH and increased the GSH/glutamate ratio (Fig. 5I). This was associated with increased glutathione peroxidase (GPX) activity in cells overexpressing HDAC4 and 5 (Fig. 5J) and reduced total H_2_O_2_ production (Fig. S4C), including from mitochondria and NADPH oxidases (Fig. 5K). To gain insights into whether overexpression of HDAC4 and 5 increases the abundance of lipids that undergo peroxidation during ferroptosis, we explored our existing lipidomic data from the bilateral HDAC4 and 5 mouse model (Fig. 2A). There were no differences in the levels of phosphatidylcholine (PC), lysophosphatidylcholine (LPC), lysophosphatidyl-ethanolamine (LPE) and phosphatidylethanolamine (PE) lipids that undergo peroxidation during ferroptosis [33] in skeletal muscle of bilateral HDAC4 and 5 mice (Fig. S4D), although many of these lipid species tended to be higher in muscle overexpressing HDAC4 and 5. Therefore, these data indicate that increased HDAC4 and 5 alters redox inputs to ferroptosis that are regulated by oxidative capacity.

### HDAC4 and 5 are required to suppress apoptosis and ferroptosis and maintain muscle mass in obese mice

**Fig. 6:**
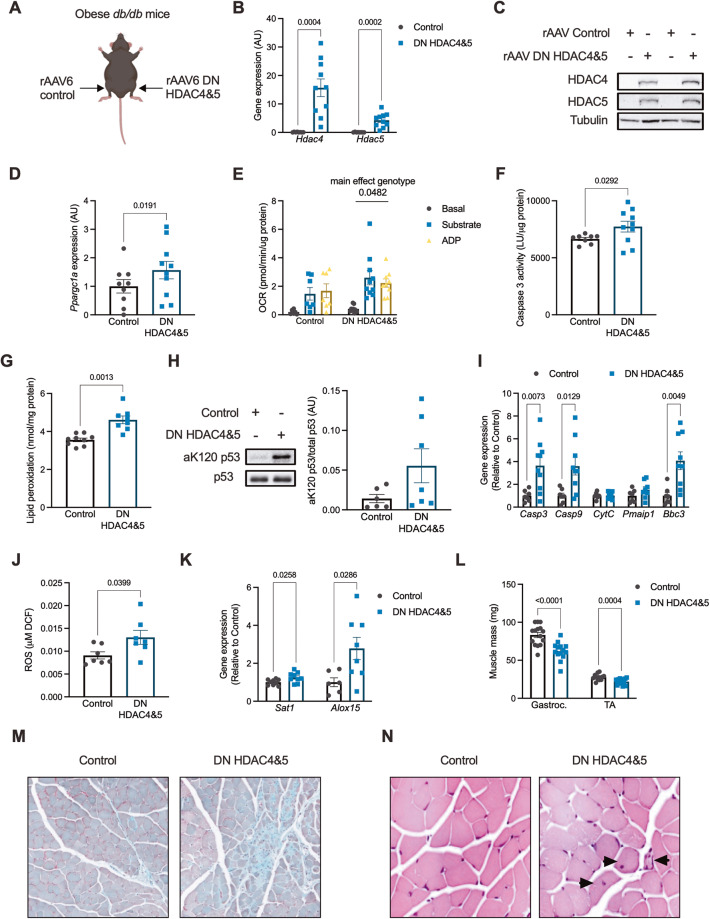
HDAC4 and 5 are required to suppress apoptosis and ferroptosis and maintain muscle mass in obese mice. **A** The bilateral AAV6 dominant negative (DN) HDAC4 and 5 overexpression *db/db* mouse model. **B** *Hdac4* and *Hdac5* gene expression in tibialis anterior (TA) skeletal muscle of bilateral AAV6 DN HDAC4 and 5 *db/db* mice (Paired *t*-tests). **C** HDAC4 and 5 protein in TA skeletal muscle of bilateral AAV6 DN HDAC4 and 5 *db/db* mice. **D** *Ppargc1a* gene expression in TA skeletal muscle of bilateral AAV6 DN HDAC4 and 5 *db/db* mice (Paired *t*-tests); (**E**) Basal, substrate and ADP-driven oxygen consumption rate (OCR) from biopsies of the gastrocnemius skeletal muscle in bilateral AAV6 DN HDAC4 and 5 *db/db* mice (Two-way ANOVA, genotype *F*(1,42) = 4.141). **F** Caspase 3 activity in TA skeletal muscle of bilateral AAV6 DN HDAC4 and 5 *db/db* mice (Paired *t*-test). **G** Lipid peroxidation in TA skeletal muscle in bilateral AAV6 DN HDAC4 and 5 *db/db* mice (Paired *t*-test). **H** Acetylated p53 at lysine 120 (aK120 p53) in TA skeletal muscle of bilateral AAV6 DN HDAC4 and 5 *db/db* mice. **I** Expression of apoptosis genes in TA skeletal muscle of bilateral AAV6 DN HDAC4 and 5 *db/db* mice (Paired *t*-tests). **J** Reactive oxygen species (ROS) in TA skeletal muscle of bilateral AAV6 DN HDAC4 and 5 *db/db* mice (Paired *t*-test). **K** Expression of ferroptosis genes in TA skeletal muscle of bilateral AAV6 DN HDAC4 and 5 *db/db* mice (Paired *t*-tests). **L** Mass of the gastrocnemius (Gastroc.) and TA skeletal muscles in bilateral AAV6 DN HDAC4 and 5 *db/db* mice (Paired *t*-tests). Staining sections of the TA muscle with (**M**) Masson’s trichrome, and; (**N**) H&E in bilateral AAV6 DN HDAC4 and 5 *db/db* mice. Arrows indicate fibres with centralised nuclei. Data are mean ± SEM, *n* = 6–10 per group.

To establish whether HDAC4 and 5 suppress apoptosis and ferroptosis in skeletal muscle in response to lipoxicity in vivo, a bilateral skeletal muscle loss-of-function HDAC4 and 5 model was developed in obese *db/db* mice using AAV6 vectors (Fig. 6A). This loss-of-function approach employed active-site HDAC4 and 5 mutants (D832N HDAC4 and D861N HDAC5), which we have previously described as acting in a dominant negative (DN) manner when overexpressed [11]. Overexpression of these mutants in skeletal muscle increases the expression of *Ppargc1a* and other metabolic genes and enhance oxidative metabolism [11]. Expression of DN HDAC4 and 5 in *db/db* mice increased HDAC4 and 5 gene (Fig. 6B) and protein (Fig. 6C) expression and increased the expression of *Ppargc1a* (Fig. 6D). Consistent with these findings, main effects for increased respiratory responses in muscle expressing DN HDAC4 and 5 were observed (Fig. 6E). Expression of DN HDAC4 and 5 increased caspase 3 activity (Fig. 6F) and lipid peroxidation (Fig. 6G), functional readouts of apoptosis and ferroptosis, respectively. Examination of the mechanisms involved in HDAC4 and 5-mediated apoptosis revealed that p53 acetylation of K120 tended to be higher (*p* = 0.08; Fig. 6H), and the expression of p53-dependent pro-apoptosis genes, including *Casp3, Casp9* and *Bbc3* was increased (Fig. 6I). The ferroptosis mechanisms regulated by HDAC4 and 5 were also examined. In contrast to observations in myogenic cells, the expression of *Slc7a11* in skeletal muscle was low and close to assay detection limits (Fig. S5A). This suggests that system x_c_ likely has only a minor role in the regulation of ferroptosis in skeletal muscle. There were no differences in GPX activity between groups (Fig. S5B). However, expression of DN HDAC4 and 5 increased ROS (Fig. 6J) and increased *Sat1* and *Alox15* expression (Fig. 6K), indicating increased capacity for lipid peroxidation. Increased apoptosis and ferroptosis in skeletal muscles expressing DN HDAC4 and 5 were associated with reduced mass of the gastrocnemius and the TA muscles (Fig. 6L). In the TA muscle, there was no difference in fibre diameter between groups (Fig. S5C). However, Masson’s trichrome staining showed sporadic areas of localised fibrosis in response to DN HDAC4 and 5 overexpression (Fig. 6M). In skeletal muscles overexpressing DN HDAC4 and 5 there were also discreet regions of fibres with centralised nuclei (Fig. 6N), which is associated with regenerating muscle fibres. These data show that HDAC4 and 5 are required to suppress apoptosis and ferroptosis and to maintain muscle mass and integrity in obese mice.

## Discussion

Lipotoxicity has a major impact on the metabolism, function and survival of various tissues including skeletal muscle. The current study has revealed that increases in HDAC4 and 5 in response to lipotoxicity induce transcriptional repression of oxidative metabolism and of the apoptosis and ferroptosis cell death pathways in both myogenic cells and skeletal muscle. The impairment in oxidative metabolism also reduces redox inputs to the ferroptosis pathway. These data indicate that metabolic reprogramming in skeletal muscle in response to lipotoxicity is directly linked with cell survival responses. These findings present an alternative paradigm for our understanding of metabolic reprogramming in chronic diseases such as obesity and type 2 diabetes, which are characterised by lipotoxicity, by suggesting that alterations in metabolism should be viewed as protective, rather than pathogenic per se. This view is consistent with a number of recent perspective pieces hypothesising this point [34–36]. These findings have important implications for the treatment of metabolic diseases, where one prevailing treatment philosophy is to increase nutrient uptake by peripheral tissues, such as skeletal muscle. Our data suggest that in the absence of enhanced energy expenditure, this approach would only place further pressure on the cell survival mechanisms identified in this study.

Previous findings revealed that lipotoxicity is associated with suppression of a pro-apoptosis transcriptional programme in skeletal muscle and maintenance of muscle mass [37]. Our data show that the class IIa HDACs are essential for this response, as loss of HDAC4 and 5 function in skeletal muscle in a model of lipotoxicity increased apoptosis genes and markers of apoptosis, which was associated with reduced muscle mass. Furthermore, a readout of ferroptosis was also increased. The exact contribution of these cell death pathways to the reduction in muscle mass observed remains to be determined. However, inhibition of ferroptosis alone enhanced cell viability following palmitate exposure while inhibition of apoptosis alone did not, possibly revealing a greater importance for ferroptosis. The role of ferroptosis in skeletal muscle pathologies is just emerging but has been implicated in the development of sarcopenia, rhabdomyolysis and inflammatory myopathies [38]. Our study also implicates skeletal muscle ferroptosis in sarcopenic obesity.

While there is much debate in the literature about the relationship between mitochondrial capacity and function and insulin action [39], the reduction in oxidative capacity induced by HDAC4 and 5 did not result in overt insulin resistance. Overexpression of HDAC4 and 5 increased basal glucose clearance, with more glucose directed towards non-oxidative pathways such as glycogen synthesis. Oxidative utilisation of glucose was impaired through the TCA cycle, particularly at the level of succinate. The levels of succinate are thought to reflect the redox state of the mitochondrial coenzyme Q pool, with higher concentrations of reduced CoQ (CoQH_2_) preventing the oxidation of succinate by SDH [40]. Oxidation of succinate by SDH drives reverse electron flow to complex I of the electron transport chain, increasing ROS production [41]. Indeed, succinate oxidation-dependent ROS production is critical for pro-inflammatory cytokine production by macrophages [42], adipose tissue thermogenesis [43] and the activity of carotid body oxygen sensing K^+^ channels [44]. In the context of the present study, HDAC4 and 5-mediated impairments in succinate oxidation and reduced ROS production likely contribute to inhibition of the ferroptosis pathway and preservation of cell viability in response to lipotoxicity. Additionally, impairments in TCA cycle activity have recently been found to promote cytosolic glutathione synthesis that increases antioxidant capacity [25], which would also inhibit ferroptosis. Together, these data indicate that inhibition of oxidative substrate utilisation is an important adaptation to lipotoxicity.

Our findings indicate that p53 is an important regulator of skeletal muscle homeostasis in the context of obesity. Best characterised as a tumour suppressor protein, p53 can regulate cell death responses, cellular senescence and cell growth in a context-dependent manner [45]. Mice with muscle-specific knockout of p53 have similar muscle mass to wild type mice and have similar reductions in muscle mass in response to denervation and aging [46, 47]. However, transgenic mice expressing a truncated form of p53 that increases its stability and activity have reduced muscle mass [48]. Furthermore, muscle atrophy in a model of cancer cachexia was attenuated in mice lacking p53 in muscle [49]. This suggests that p53 reduces muscle mass in certain circumstances, including in obesity. Interestingly, a polymorphism in the p53 gene (*TP53*) that enhances p53-mediated apoptosis increases susceptibility to sarcopenic obesity [50]. Additional research will be required to further understand the role of p53 in muscle in obesity. For example, the relative role of p53 in mature myofibres and muscle satellite cells, which influence the muscle regenerative response, could not be examined in our in vivo investigations and is a limitation of our study. Nonetheless, discovery that the apoptosis functions of p53 can be impaired by the class IIa HDACs in response to lipotoxicity also has implications for certain cancers, particularly those associated with obesity.

In conclusion, this study provides evidence that in response to lipotoxicity the class IIa HDACs unite impairments in skeletal muscle oxidative metabolism with inhibition of the apoptosis and ferroptosis cell death pathways to preserve muscle integrity. These findings provide key new insights into why certain metabolic adaptations occur in response to excess lipids and advance our understanding of the aetiology of metabolic diseases driven by lipotoxicity and excess nutrient availability.

### Supplementary information

Supplementary information

Original Data File

## Data Availability

The microarray datasets are available at Gene Expression Omnibus (GSE215185).
