# Supplementary material for: Class IIa HDACs inhibit cell death pathways and protect muscle integrity in response to lipotoxicity
Source: Cell Death Dis. 2023 Dec 1;14(12):787. doi: 10.1038/s41419-023-06319-5 (PMC10692215; doi:10.1038/s41419-023-06319-5)
Supplement: Supplementary file 1 — Supplementary information [file 41419_2023_6319_MOESM1_ESM.docx]

**Supplementary Information for**

**Class IIa HDACs inhibit cell death pathways and protect muscle integrity in response to lipotoxicity**

Sheree D. Martin^1*^, Timothy Connor^1*^, Andrew Sanigorski^1^, Kevin A. McEwen^1^, Darren C. Henstridge^2,3^, Brunda Nijagal^4^, David De Souza^4^, Dedreia L. Tull^4^, Peter J. Meikle^3^,

Greg M. Kowalski^1,5^, Clinton R. Bruce^5^, Paul Gregorevic^6^, Mark A. Febbraio^7^, Fiona M. Collier^8^, Ken R. Walder^1^, Sean L. McGee^1^

^1^Institute for Mental and Physical Heath and Clinical Translation (IMPACT) and Metabolic Research Unit, School of Medicine, Deakin University, Geelong, Victoria 3216, Australia; ^2^College of Health and Medicine, School of Health Sciences, University of Tasmania, Launceston, Australia;

^3^Baker Heart and Diabetes Institute, Melbourne, Victoria 3004, Australia;

^4^Metabolomics Australia, The University of Melbourne, Parkville, Victoria 3010, Australia; ^5^Institute of Physical Activity and Nutrition (IPAN) and School of Exercise and Nutrition Sciences, Deakin University, Geelong, Victoria 3216, Australia;

^6^Centre for Muscle Research, Department of Anatomy and Physiology, The University of Melbourne, Parkville, VIC, Australia.

^7^Monash Institute of Pharmaceutical Sciences, Monash University, Parkville, VIC, Australia.

^8^Barwon Health, Geelong, Victoria 3220, Australia

^*^These authors contributed equally to this work

Corresponding author:

Professor Sean L. McGee

Metabolic Remodelling Laboratory

Metabolic Research Unit

School of Medicine

Deakin University, Waurn Ponds, Australia, 3216

Ph: +61 3 5227 2519

Fax: +61 3 5227 2945

Email: sean.mcgee@deakin.edu.au

**TABLE S1: Real time RT-PCR primers**

Gene Primer Sequence (5’-3’)

*Aifm1* Forward CAGAGAAGAGCCATTGCCTCC

Reverse ATACAATCAGGACCCTGGCCCC

*Aifm2* Forward ACTCCTTCCACCACAATG

Reverse CGGTTCTTCAAGTCTATGC

*Alox15* Forward AGGGCTGGGGCTAATTAGGA

Reverse TGCGCAGTGAGCTAGTGAAA

*Apaf1* Forward CTTCTTTATGGTGCTGAAGATTGA

Reverse GGTGGAGTGCCTGTCTAGTGT

*Bax* Forward ATGGAGCTGCAGAGGATGAT

Reverse GAAGTTGCCATCAGCAAACA

*Bbc3* Forward AGACAAGAAGAGCAGCATCGACAC

Reverse TAGGCACCTAGTTGGGCTCCATTT

*Bcl2* Forward ACGCTCTCCACACACATGAC

Reverse GGTGGTGGAGGAACTCTTCA

*Bcl2l1* Forward TGAATGACCACCTAGAGCCTTG

Reverse CAGAACCACACCAGCCACAG

*Casp3* Forward TGGACTGTGGCATTGAGACAG

Reverse CGACCCGTCCTTTGAATTTC

*Casp9* Forward GGATGCTGTGTCAAGTTTGCC

Reverse CTTTCGCAGAAACAGCATTGG

*Cox7a1* Forward CAGCGTCATGGTCAGTCTGT

Reverse AGAAAACCGTGTGGCAGAGA

*Cpt1b* Forward TCGCAGGAGAAAACACCATGT

Reverse AACAGTGCTTGGCGGATGTG

*Cs* Forward GCCAGATCACTGTGGACATGAT

Reverse CAAGAACCGAAGTCTCATACACAAG

*CytC* Forward CATCCCTTGACATCGTGCTT

Reverse GGGTAGTCTGAGTAGCGTCGTG

*Echs1* Forward ATGGAGATGGTCCTCACTGG

Reverse CGCCATGGCTACTACGATTT

*Fh* Forward AATGACACCTTTCCCACAGC

Reverse GCTTCTGTAACCCTGGCAAC

*Gsl2* Forward CTTAGGCACTGACTACGTGCACAAG

Reverse CCCACAGATCTCCACTATATGCAGG

*Hadh* Forward ACCAAACGGAAGACATCCTG

Reverse AGCTCAGGGTCTTCTCCACA

*Hdac4*  Forward CCATGAAGCACCAGCAGGAG

Reverse CTCGCCACAGCACTCTCTTTG

*Hdac5*  Forward TCGCTGAGAACGGCTTTACTGG

Reverse ATGTTGGGCAGAGAAGGAGACG

*Idh1* Forward GTTGGTCTTCACCCCAAAGA

Reverse GAAATGGACTCGTCGGTGTT

*Mcad* Forward GCTAGTGGAGCACCAAGGAG

Reverse CCAGGCTGCTCTCTGGTAAC

*Mcl1* Forward AGCACATTTCTGATGCCGCCT

Reverse GTGCCTTTGTGGCCAAACACT

*Pmaip1*  Forward GCTACCACCTGAGTTCGC

Reverse TCGTCCTTCAAGTCTGCTG

*Ppargc1a* Forward CCCTGCCATTGTTAAGACC

Reverse TGCTGCTGTTCCTGTTTTC

*Ppard* Forward CACTTGTTGCGGTTCTTCTTC

Reverse CCTCGGGCTTCCACTACG

*Sat1* Forward AAGTGTCGCTGCAGTAT

Reverse AGCCTCCATCCCTGTTCACT

*Slc7a11* Forward TGGCGGTGACCTTCTCTGA

Reverse ACAAAGATCGGGACTGCTAATGA

**TABLE S2: Antibodies**

Antibody Company Dilution

HDAC4 Cell Signaling 1:1000 in TBST

HDAC5 Santa Cruz 1:1000 in TBST

OXPHOS cocktail MitoSciences 1:1000 in TBST

pT308 Akt Cell Signaling 1:1000 in TBST

pS473 Akt Cell Signaling 1:1000 in TBST

Akt Cell Signaling 1:1000 in TBST

Caspase 3 Cell Signaling 1:1000 in TBST

Cleaved caspase 3 Cell Signaling 1:1000 in TBST

Caspase 9 Cell Signaling 1:1000 in TBST

Cleaved caspase 9 Cell Signaling 1:1000 in TBST

acetyl K120 p53 Abcam 1:500 in TBST

p53 Cell Signaling 1:1000 in TBST

pS642 TBC1D4 Cell Signaling 1:1000 in TBST

TBC1D4 Cell Signaling 1:500 in 1% BSA in TBST

α-tubulin Sigma-Aldrich 1:1000 in TBST

**SUPPLEMENTARY FIGURE 1**

**
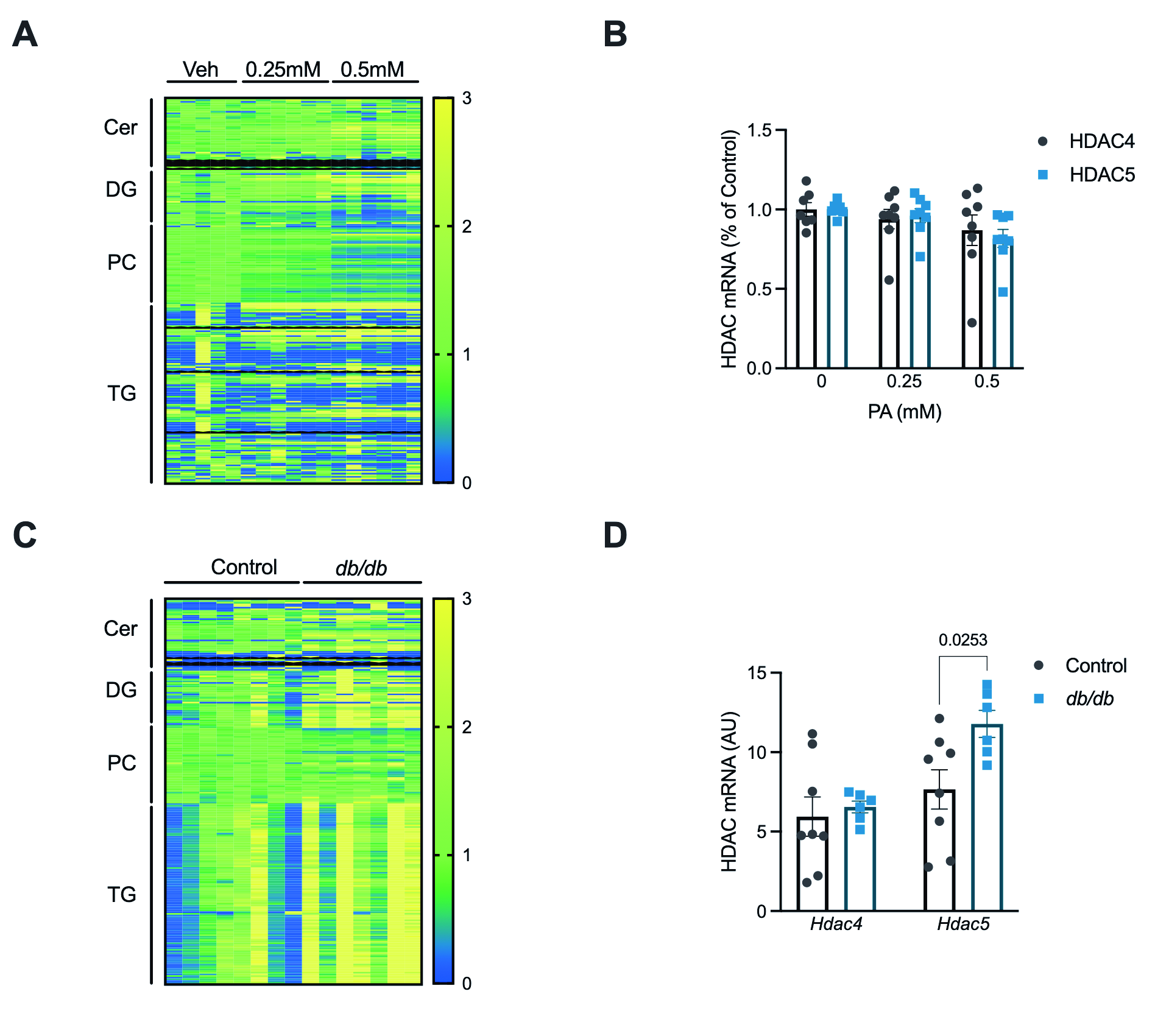
**

**Supplementary Figure 1:** (A) Heat map of ceramide (Cer), diglyceride (DG), phosphatidylcholine (PC) and triglyceride (TG) lipid species in C2C12 myotubes treated with 0mM (BSA vehicle), 0.25mM or 0.5mM palmitate (PA) for 16 hrs. (B) *Hdac4* and *Hdac5* gene expression following exposure to vehicle, 0.25mM or 0.5mM PA for 16hrs. (C) Heat map of Cer, DG, PC and TG lipid species in tibialis anterior (TA) skeletal muscle of Control and *db/db* mice. (D) *Hdac4* and *Hdac5* gene expression in TA skeletal muscle of Control and *db/db* mice (Unpaired t-test). Data are mean±SEM, n=6-8 biological replicates per group for cell experiments and 6-8 per group for animal experiments.

**SUPPLEMENTARY FIGURE 2**

**
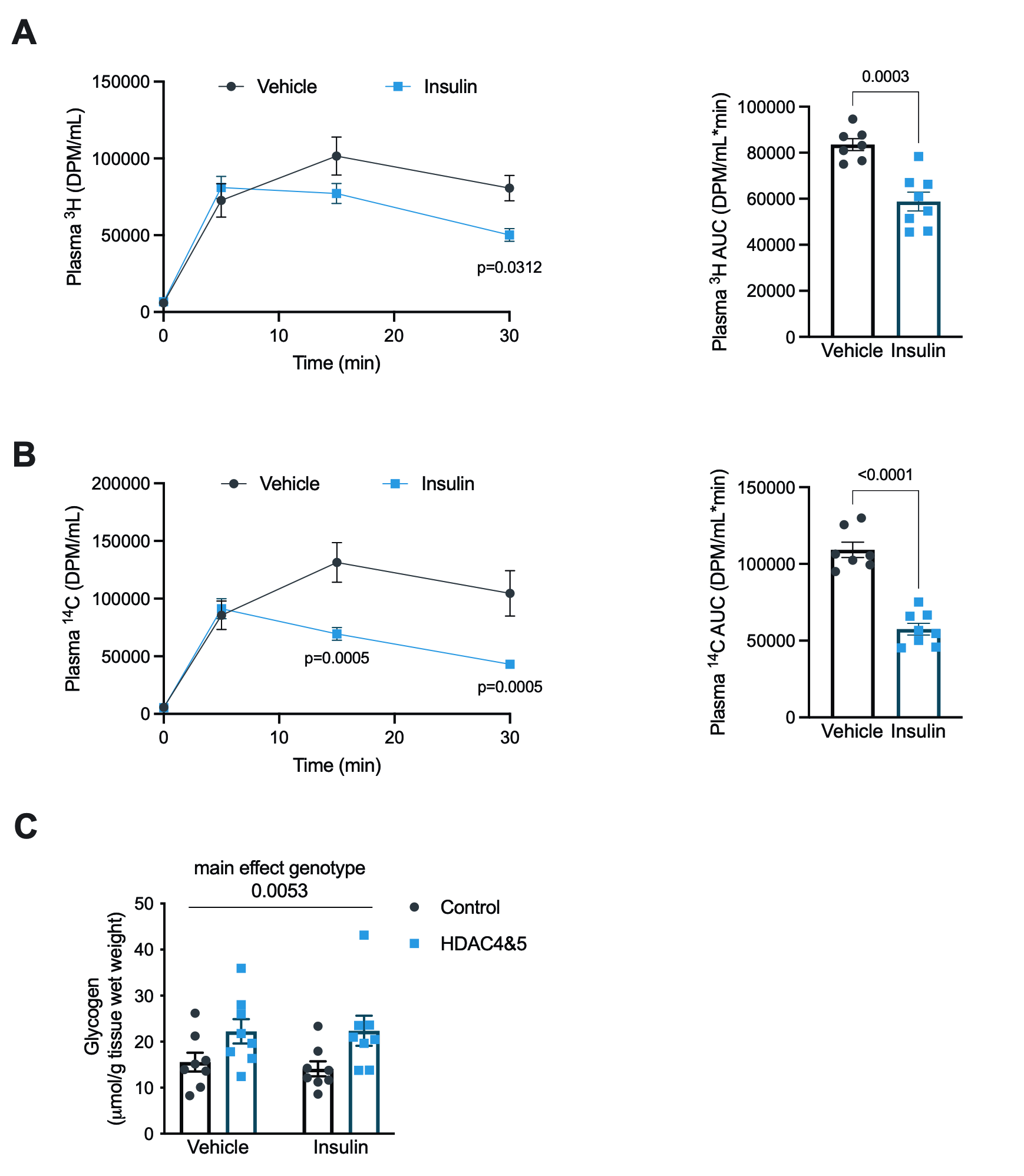
**

**Supplementary Figure 2:** (A) Plasma ^3^H at 0, 5, 15 and 30 min (Two-way ANOVA, time *p*<0.0001 *F*(2.607,36.5)=68.08, interaction *p*=0.0079 *F*(3,42)=4.511, significant Tukey’s multiple comparisons shown) and plasma ^3^H area-under-the-curve (AUC; Unpaired t-test) in bilateral AAV6 HDAC4 and 5 mice administered vehicle or insulin and isotopic glucose tracers. (B) Plasma ^14^C at 0, 5, 15 and 30 min (Two-way ANOVA, time *p*<0.0001 *F*(3,21)=47.84, treatment *p*=0.0442 *F*(1,7)=5.997, interaction *p*=0.0010 *F*(3,21)=7.944, significant Tukey’s multiple comparisons shown) and plasma ^14^C AUC (Unpaired t-test *p*<0.0001) in bilateral AAV6 HDAC4 and 5 mice administered vehicle or insulin and isotopic glucose tracers. (C) Glycogen (Two-way ANOVA, genotype *F*(1,28)=9.131) in tibialis anterior skeletal muscle bilateral AAV6 HDAC4 and 5 mice administered vehicle or insulin and isotopic glucose tracers. Data are mean±SEM, n=6-8 per group.

**SUPPLEMENTARY FIGURE 3**

**
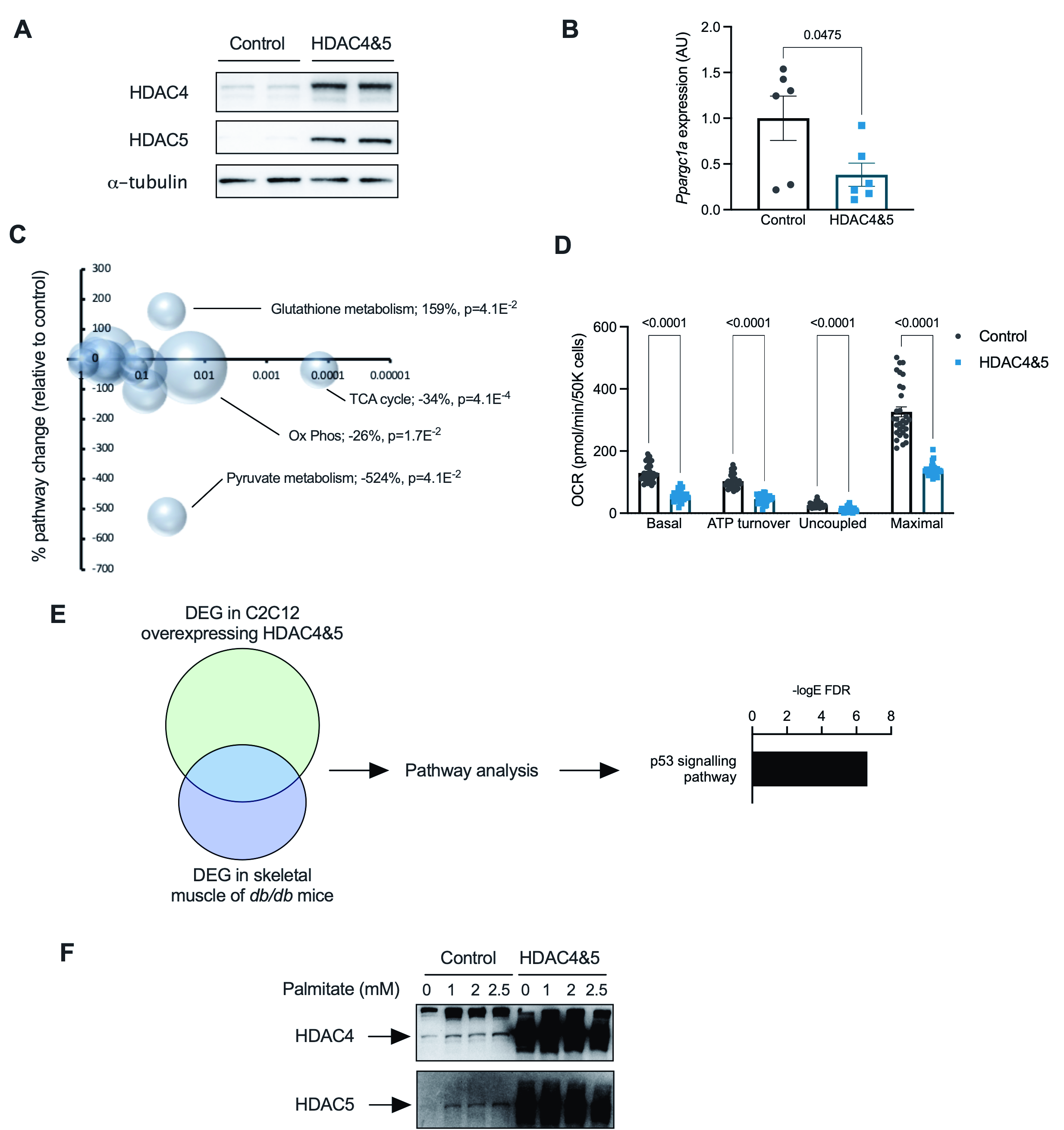
**

**Supplementary Figure 3:** (A) HDAC4 and 5 protein from two biological replicates of Control and HDAC4 and 5 overexpressing C2C12 myoblasts. (B) *Ppargc1a* gene expression in Control and HDAC4 and 5 overexpressing C2C12 myoblasts (Unpaired t-test). (C) Bubble plot of metabolic pathway gene expression in HDAC4 and 5 overexpressing C2C12 myoblasts relative to Control. (D) Oxygen consumption rate (OCR) linked to basal, ATP turnover, uncoupled and maximal respiration in Control and HDAC4 and 5 overexpressing C2C12 myoblasts (Unpaired t-tests). (E) Identification of differentially expressed genes (DEG) in skeletal muscle from *db/db* mice and C2C12 myoblasts overexpressing HDAC4 and 5 (n=3/group for skeletal muscle and cell analyses) followed by pathway enrichment analysis revealed the p53 signalling pathway as being significantly enriched in this geneset. Data are mean±SEM, n=6-20 biological replicates per group. # p<0.05 vs Control. (F) Overexposed western blots showing the increase in endogenous HDAC4 and 5 with high concentrations of palmitate in Control and HDAC4 and 5 overexpressing C2C12 myoblasts.

**SUPLEMENTARY FIGURE 4**

**
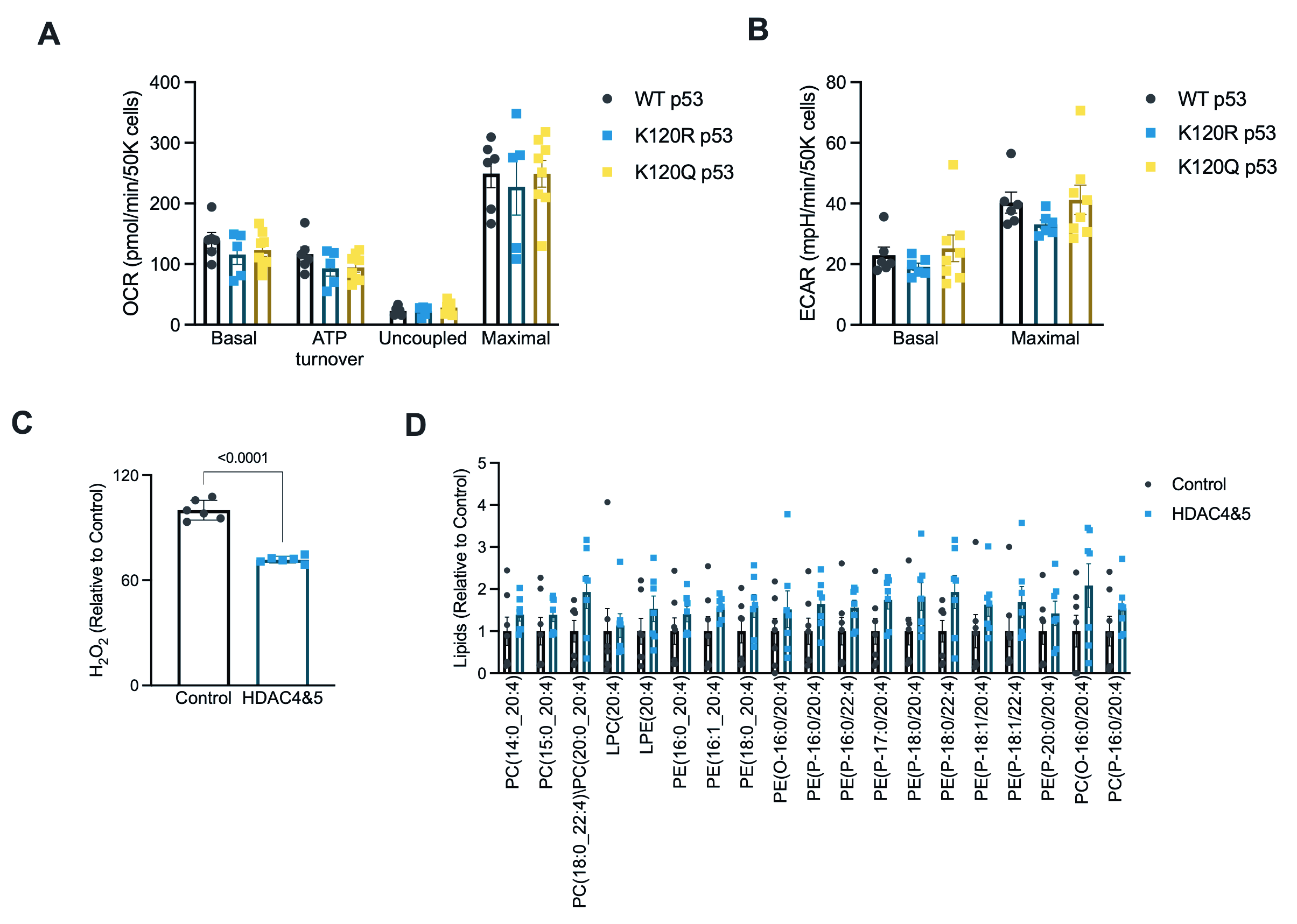
**

**Supplementary Figure 4:** (A) Oxygen consumption rate (OCR) linked to basal, ATP turnover, uncoupled and maximal respiration and (B) Basal and maximal extracellular acidification rate (ECAR) in C2C12 myoblasts expressing WT, K120R or K120Q p53. (C) Total H_2_O_2_ release from Control and HDAC4 and 5 overexpressing C2C12 myoblasts (Unpaired t-test). (D) Phosphatidylcholine (PC), lysophosphatidylcholine (LPC), lysophosphatidyl-ethanolamine (LPE) and phosphatidylethanolamine (PE) lipids in Control or HDAC4 and 5 overexpressing skeletal muscle. Data are mean±SEM, n=6 biological replicates per group for cell experiments and n=7 per group in mouse experiments.

**SUPPLEMENTARY FIGURE 5**

**
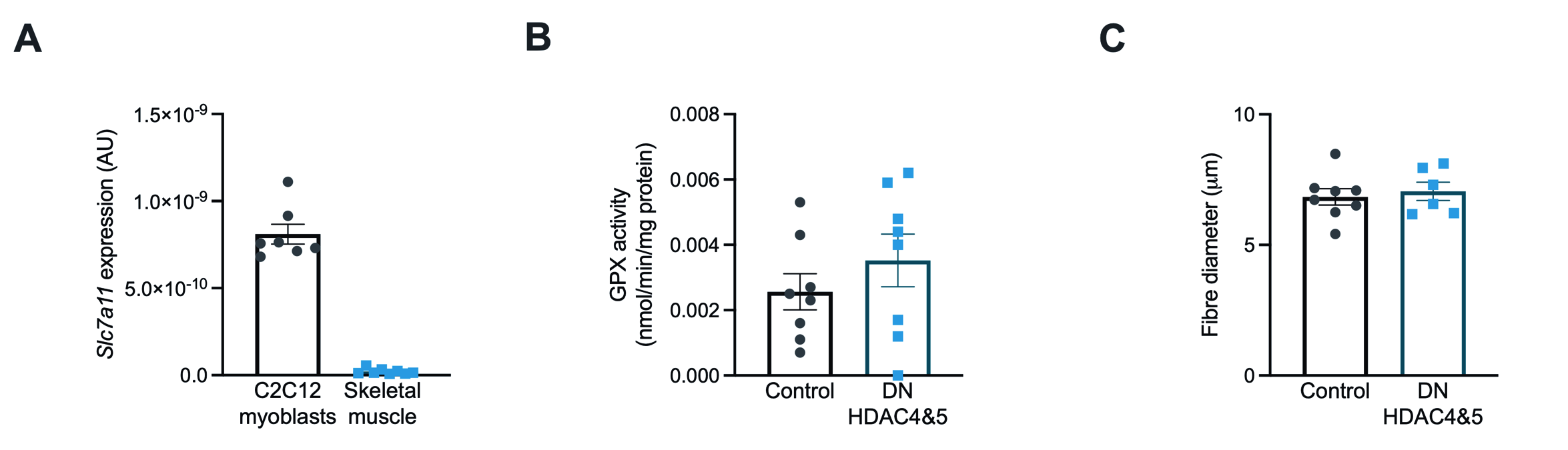
**

**Supplementary Figure 5:** (A) *Slc7a11* expression in C2C12 myoblasts or mouse skeletal muscle. (B) Glutathione peroxidase (GPX) activity, and; (C) Fibre diameter in Control and DN HDAC4 and 5 skeletal muscle of *db/db* mice. Data are mean±SEM, n=7-8 per group.
