# Supplementary material for: Class IIa HDACs inhibit cell death pathways and protect muscle integrity in response to lipotoxicity
Source: Cell Death Dis. 2023 Dec 1;14(12):787. doi: 10.1038/s41419-023-06319-5 (PMC10692215; doi:10.1038/s41419-023-06319-5)

Figure 1D

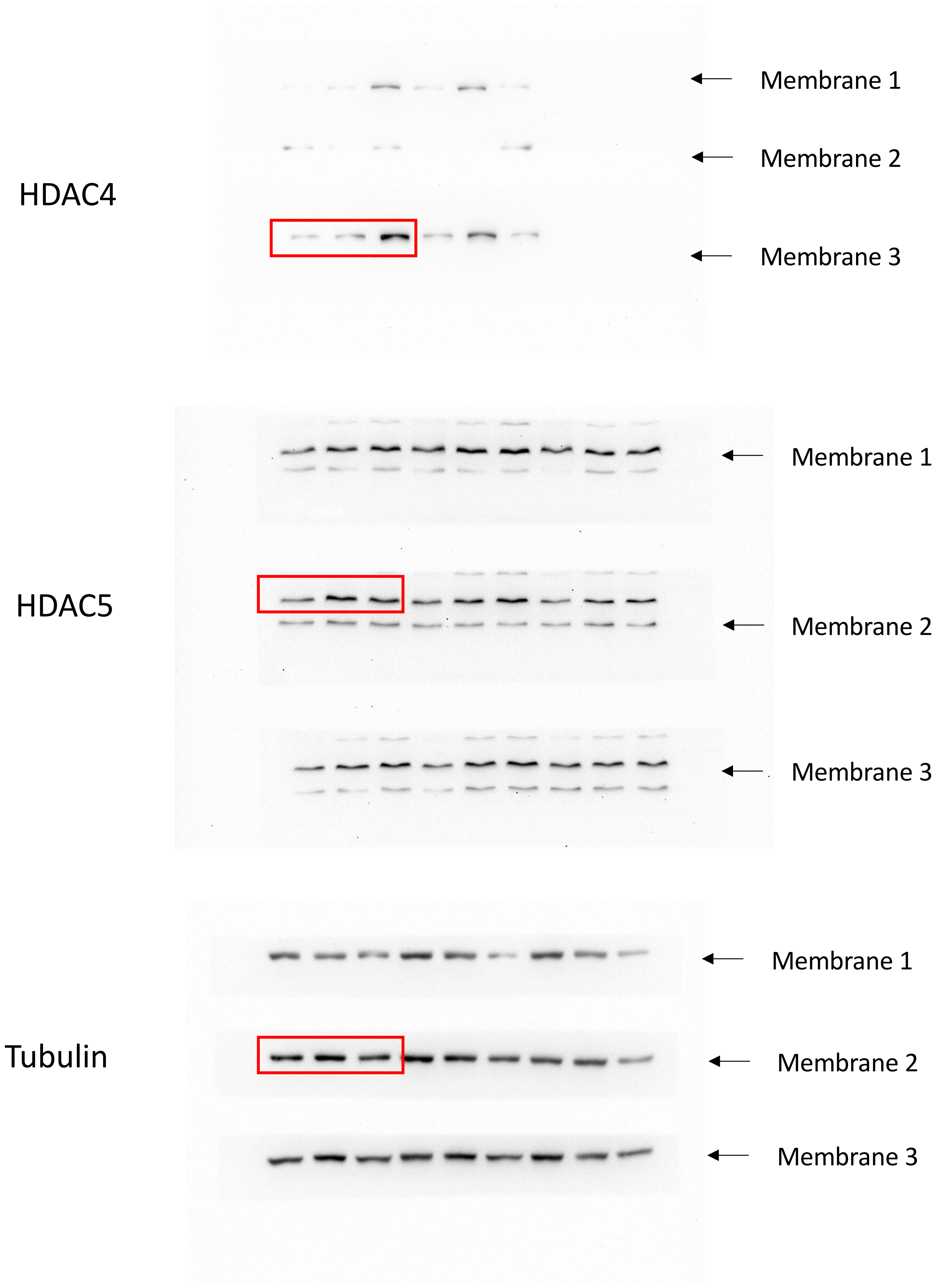
HDAC4
Membrane 1
Membrane 2
Membrane 3
HDAC5
Membrane 1
Membrane 2
Membrane 3
Tubulin
Membrane 1
Membrane 2
Membrane 3

Figure 1H

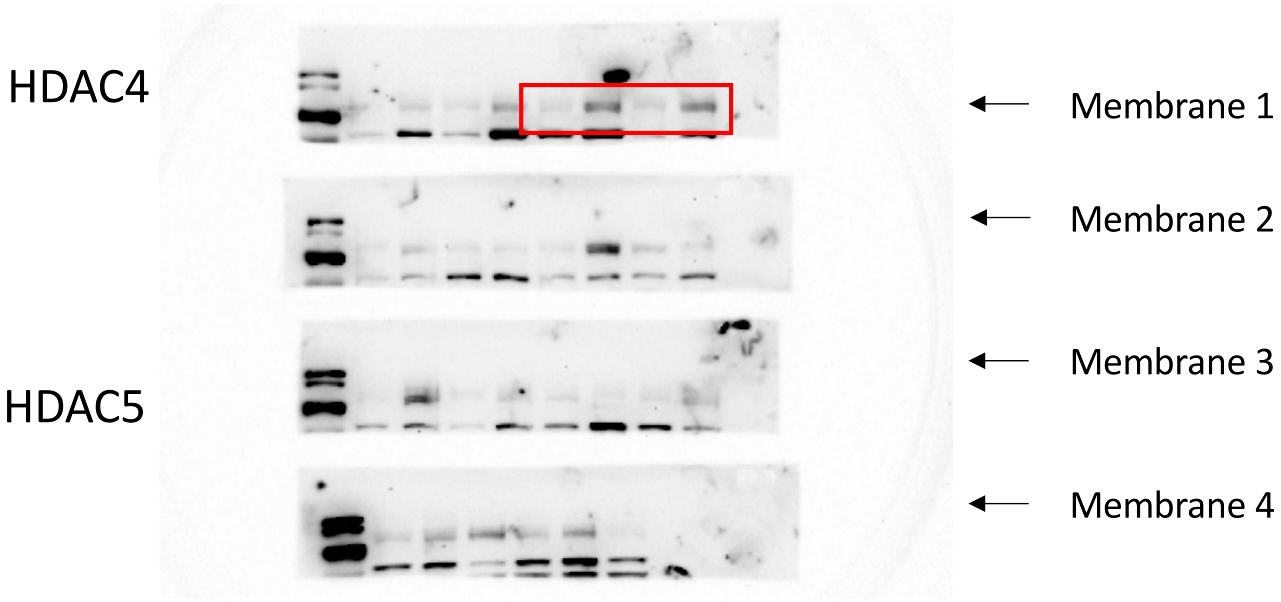
HDAC4
HDAC5
Membrane 1
Membrane 2
Membrane 3
Membrane 4

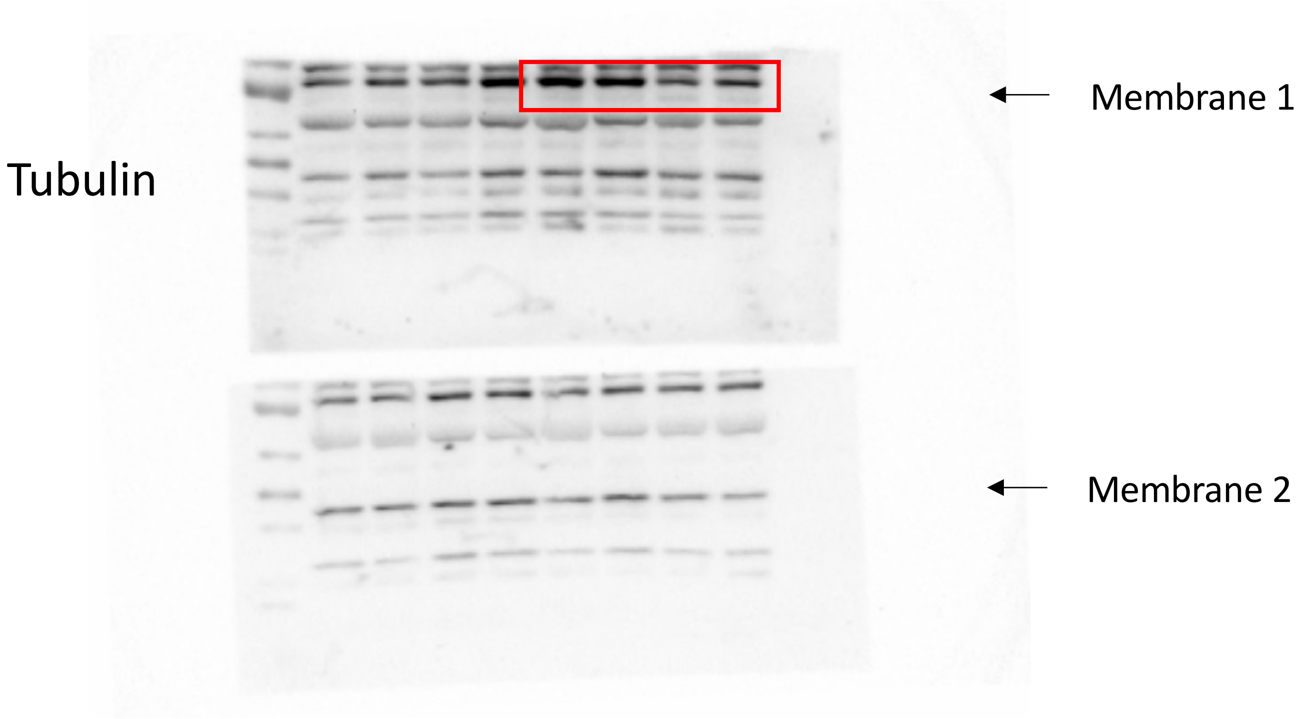
Tubulin
Membrane 1
Membrane 2

Figure 2C

HDAC4

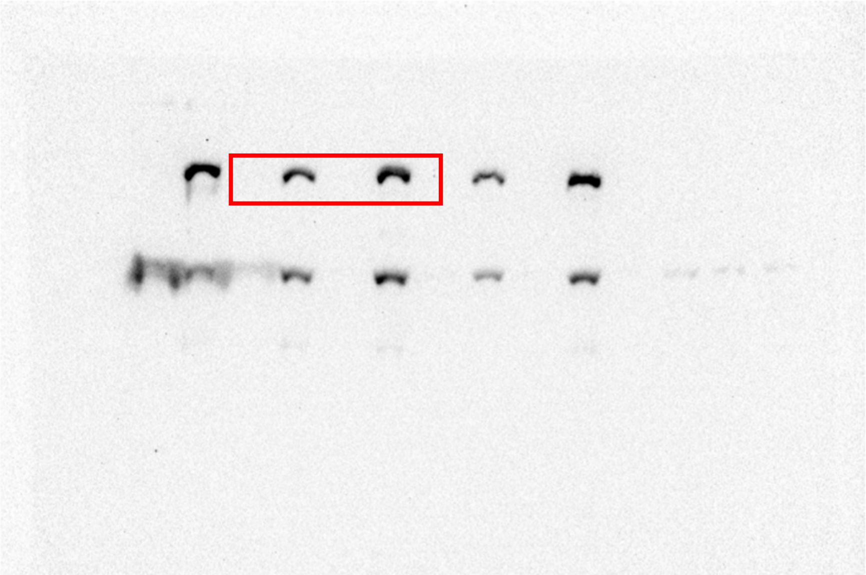

HDAC5

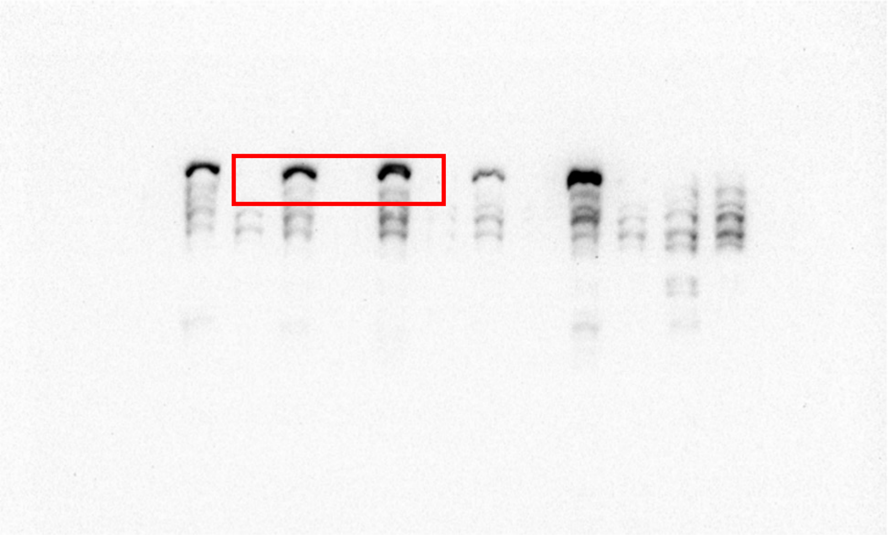

Tubulin

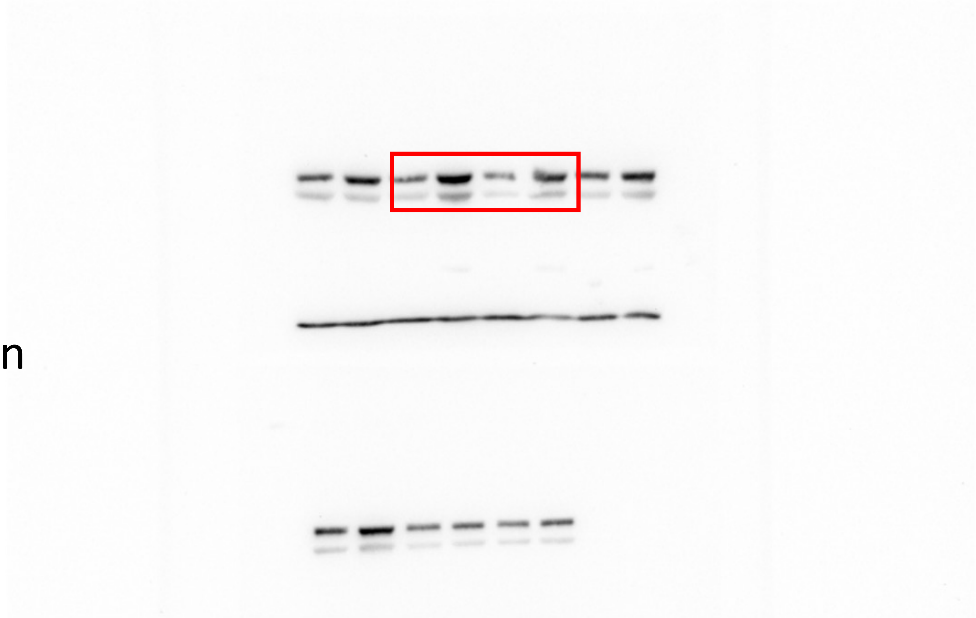

Figure 2F

Ox phos

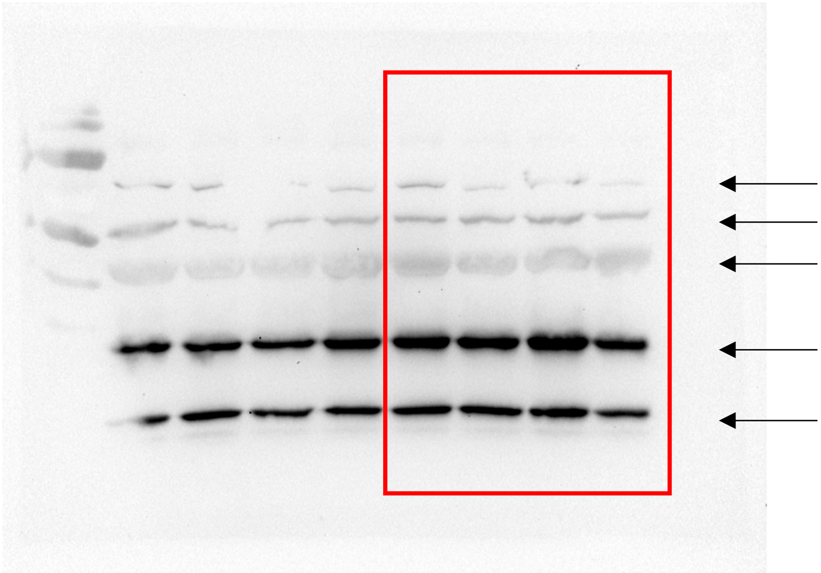

Tubulin

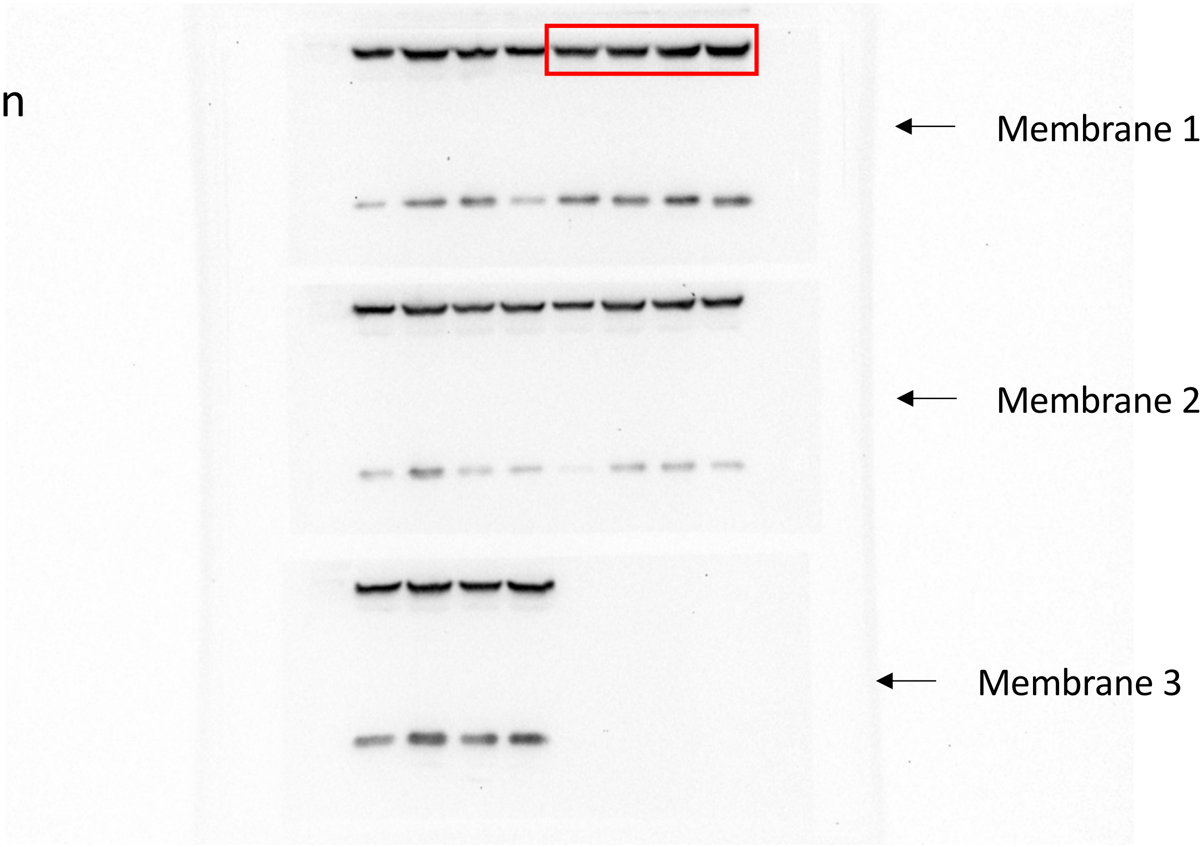

Figure 3F

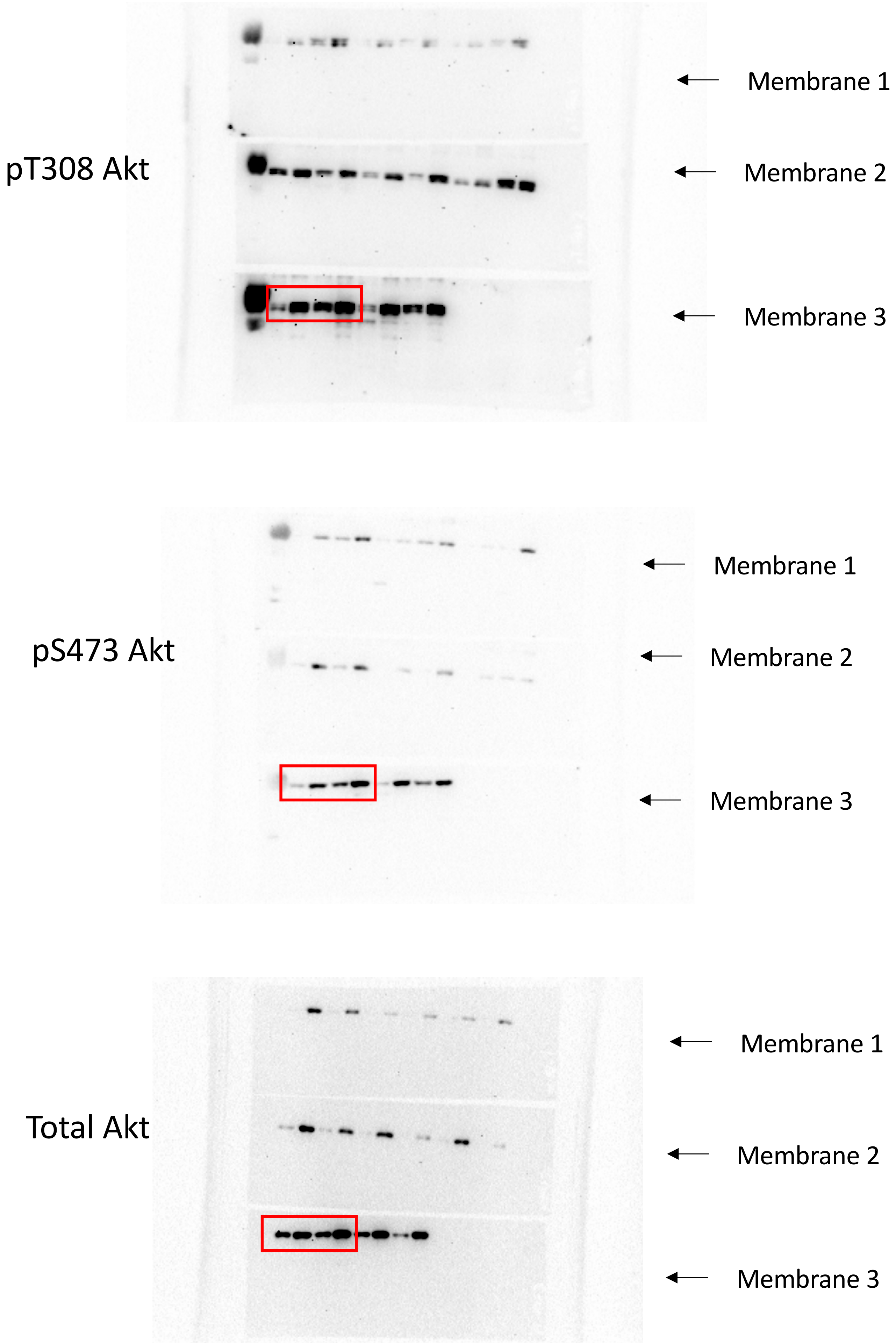
pT308 Akt
Membrane 1
Membrane 2
Membrane 3
pS473 Akt
Membrane 1
Membrane 2
Membrane 3
Total Akt
Membrane 1
Membrane 2
Membrane 3

Figure 3F

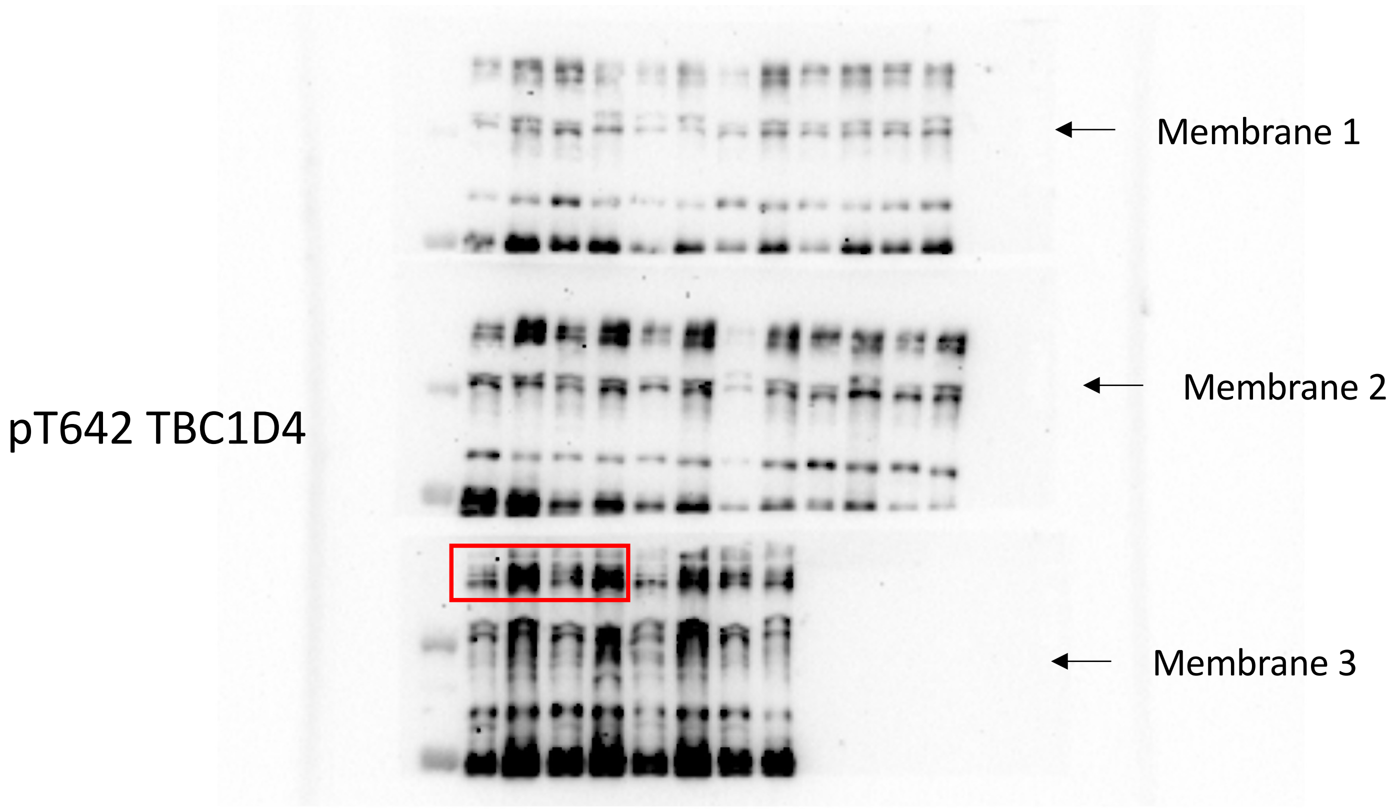
pT642 TBC1D4
Membrane 1
Membrane 2
Membrane 3

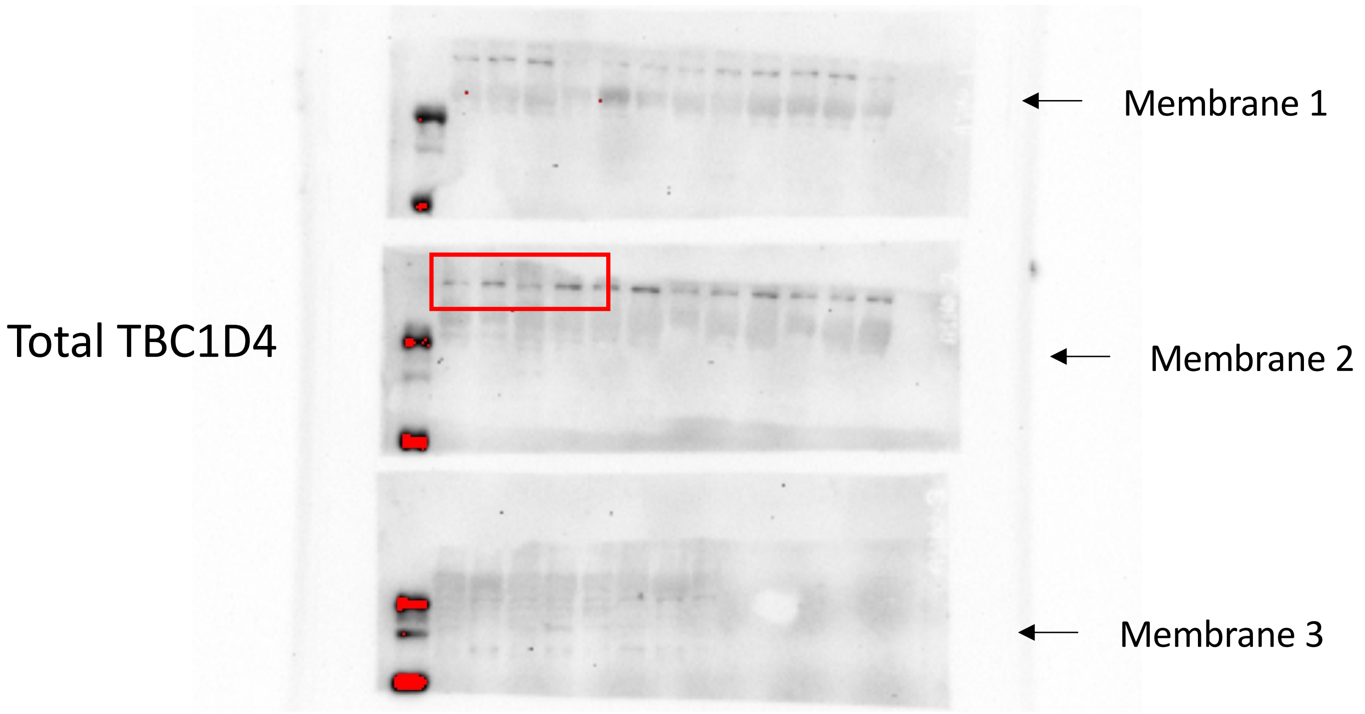
Total TBC1D4
Membrane 1
Membrane 2
Membrane 3

Figure 3F

HDAC4

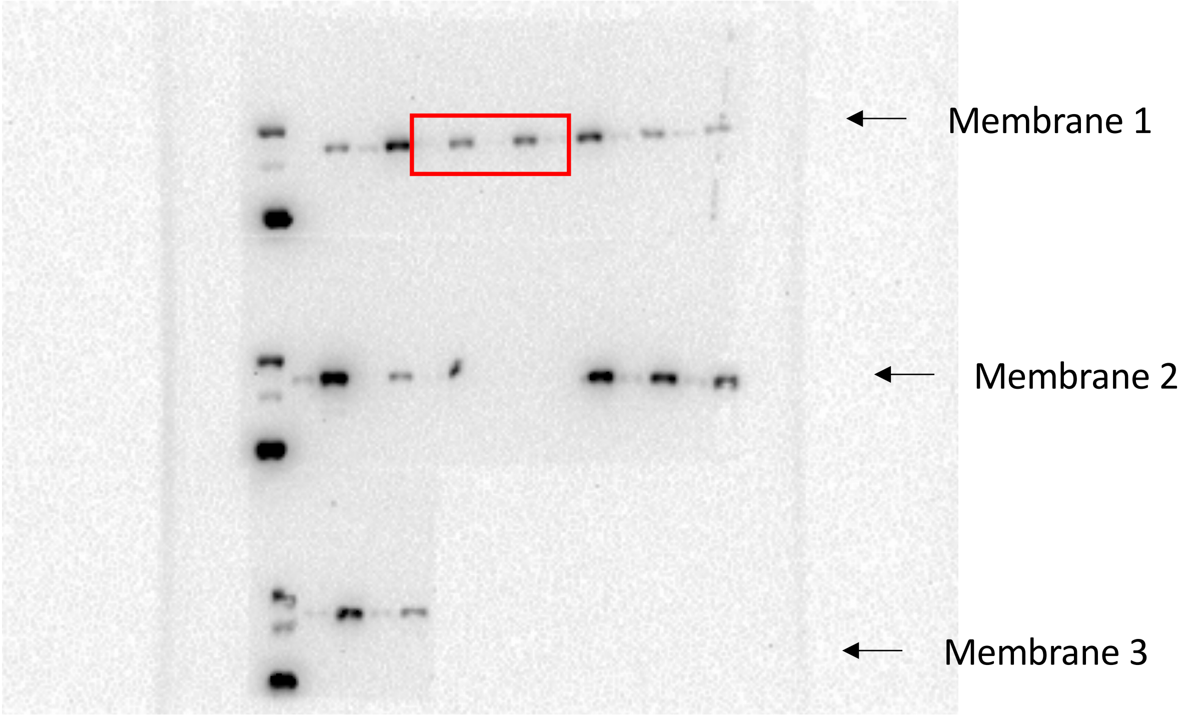

HDAC5

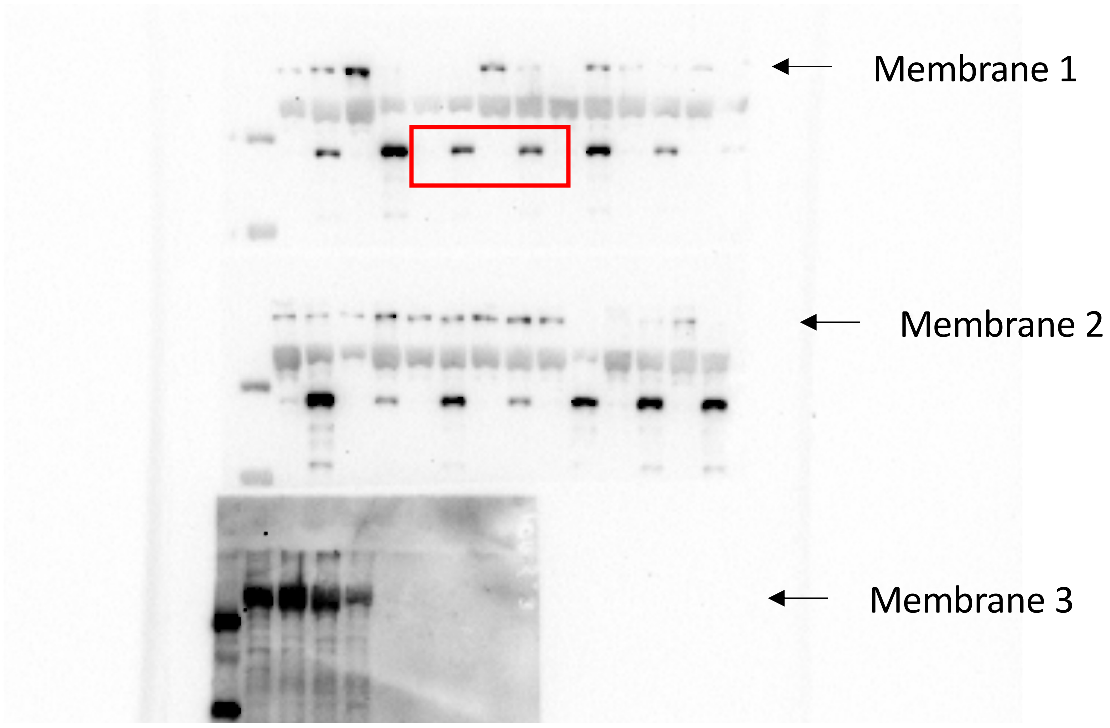

Figure 4E

Caspase 3

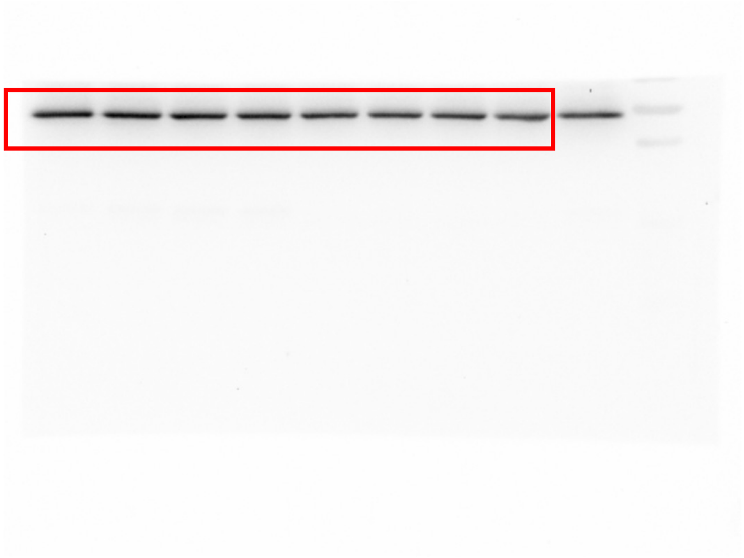

Cleaved
caspase 3

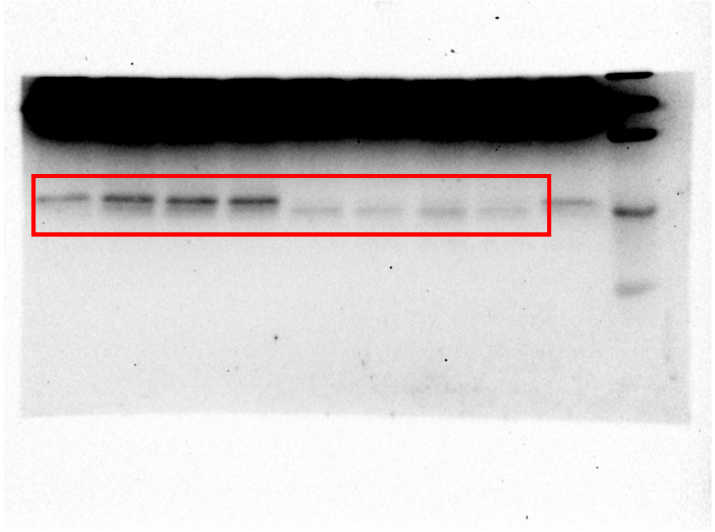

Full length (FL) and cleaved caspase 9

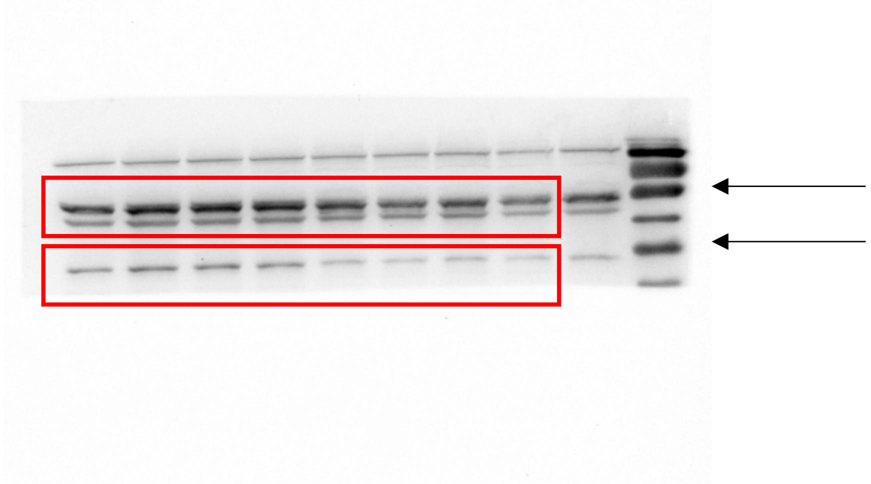

FL

Cleaved

Figure 4E

HDAC4

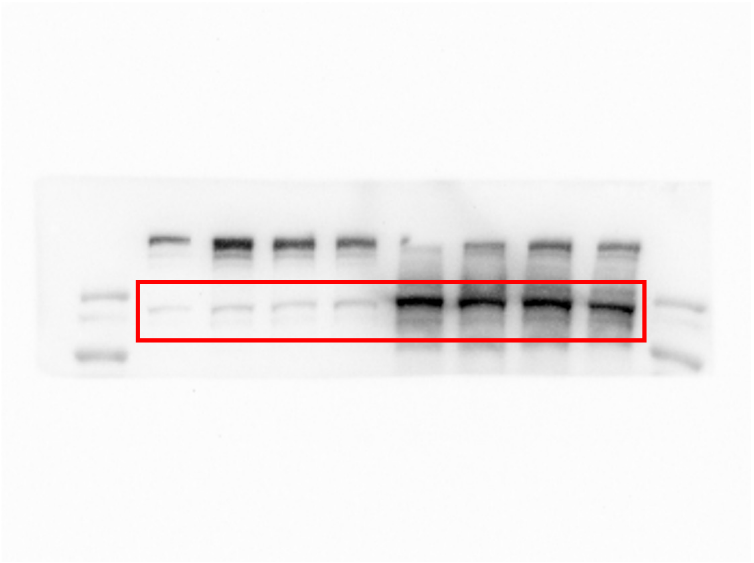

HDAC5

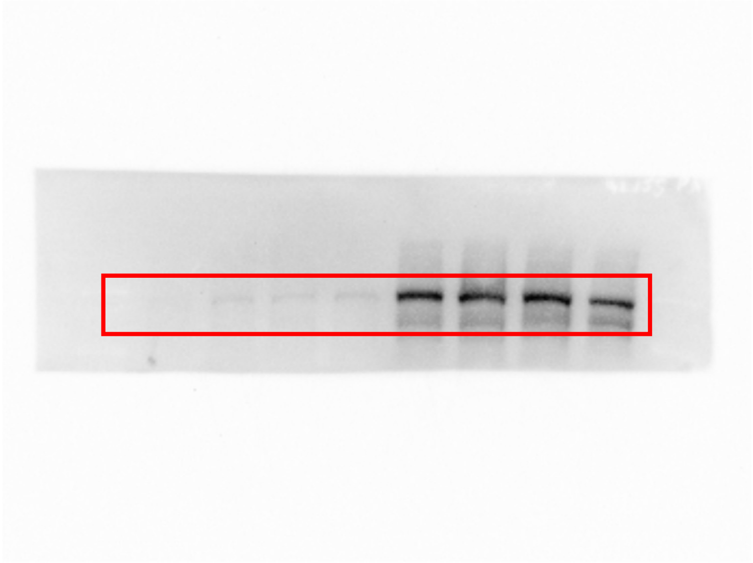

Tubulin

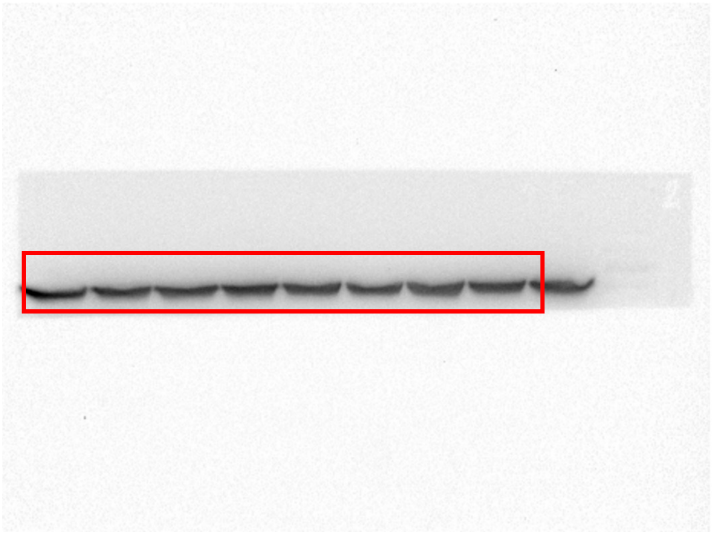

Figure 5A

aK120 p53

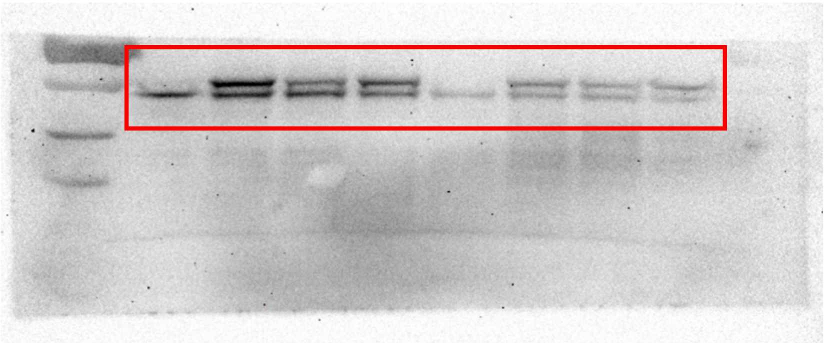

p53

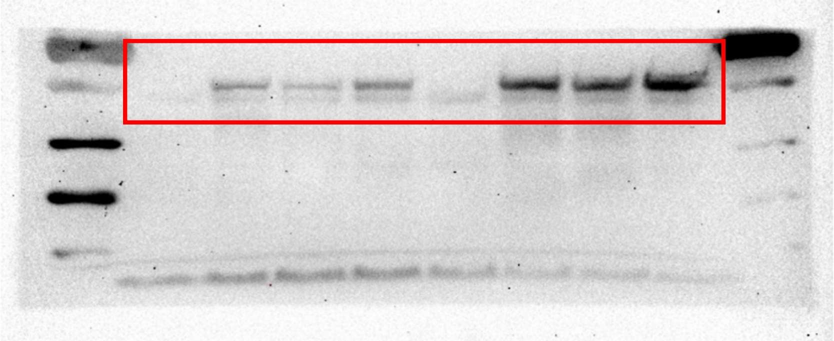

HDAC4

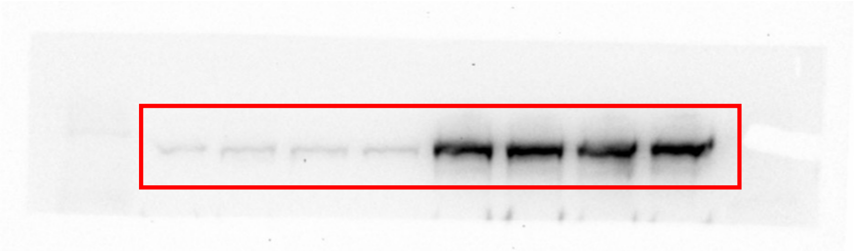

HDAC5

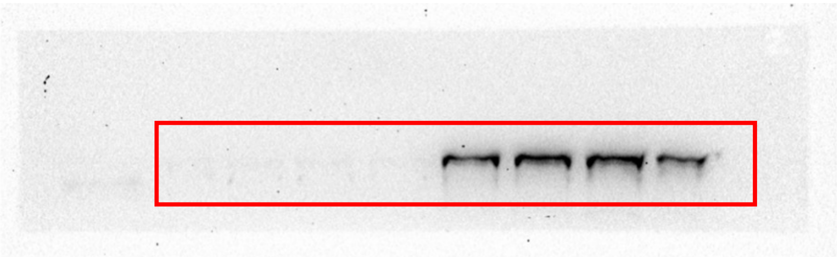

Tubulin

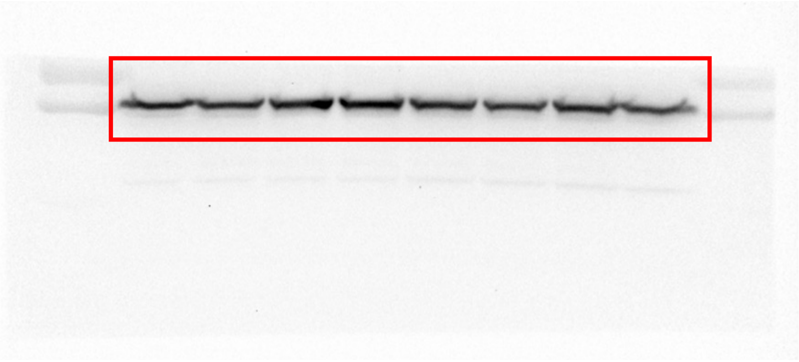

Figure 6C

HDAC4

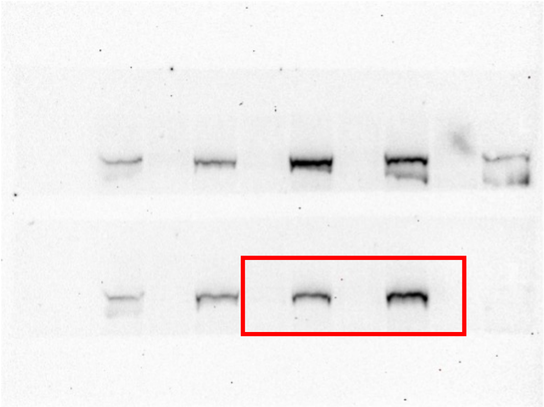

Membrane 1

Membrane 2

HDAC5

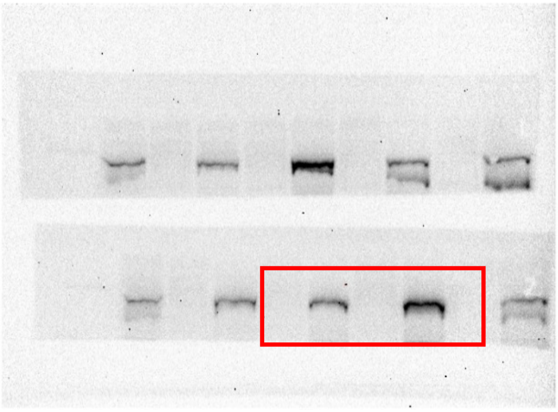

Membrane 1

Membrane 2

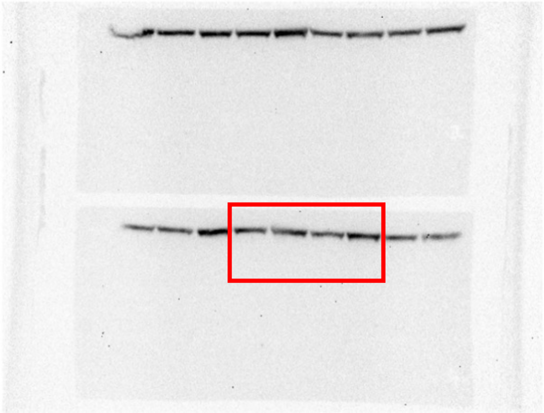

Membrane 1

Membrane 2

Tubulin

Figure 6H

aK120 p53

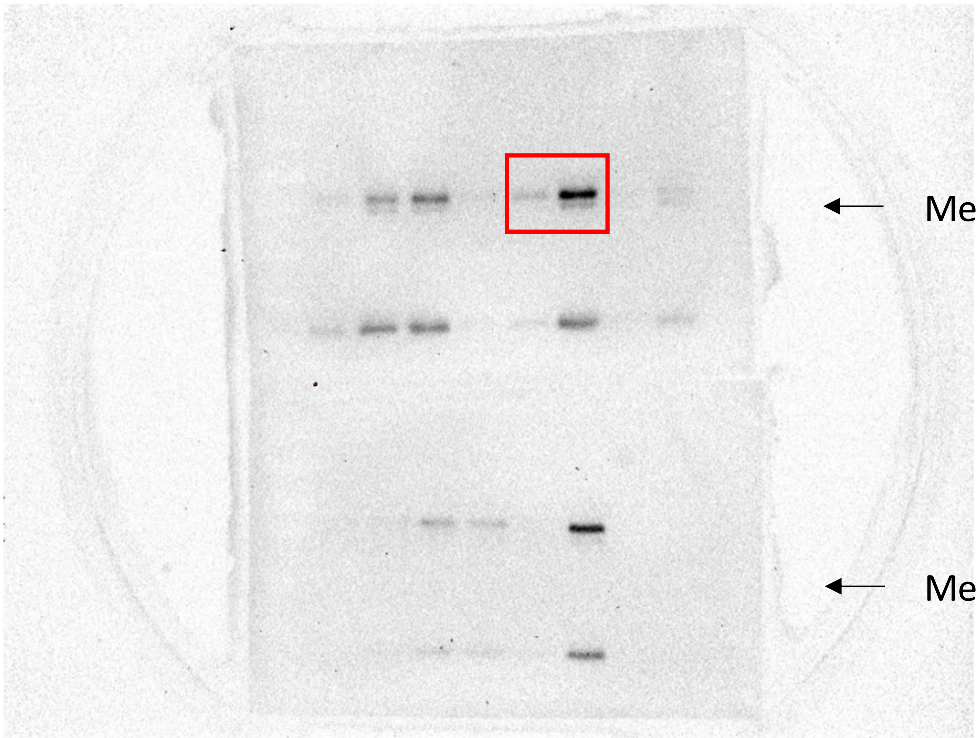

Membrane 1

Membrane 2

Total p53

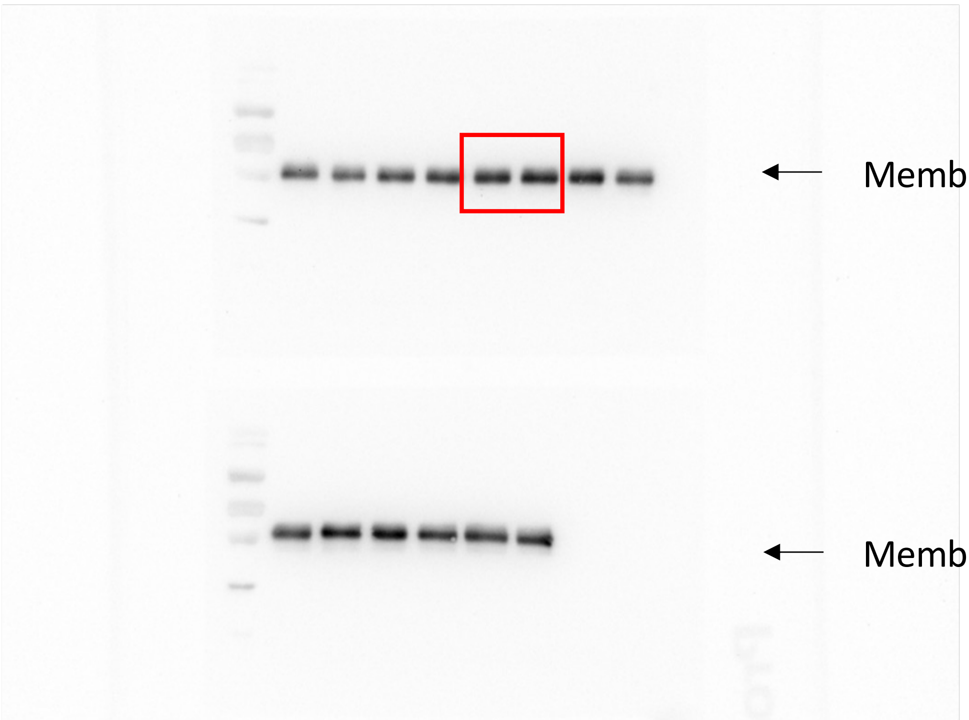

Membrane 1

Membrane 2

Figure S3A

HDAC4

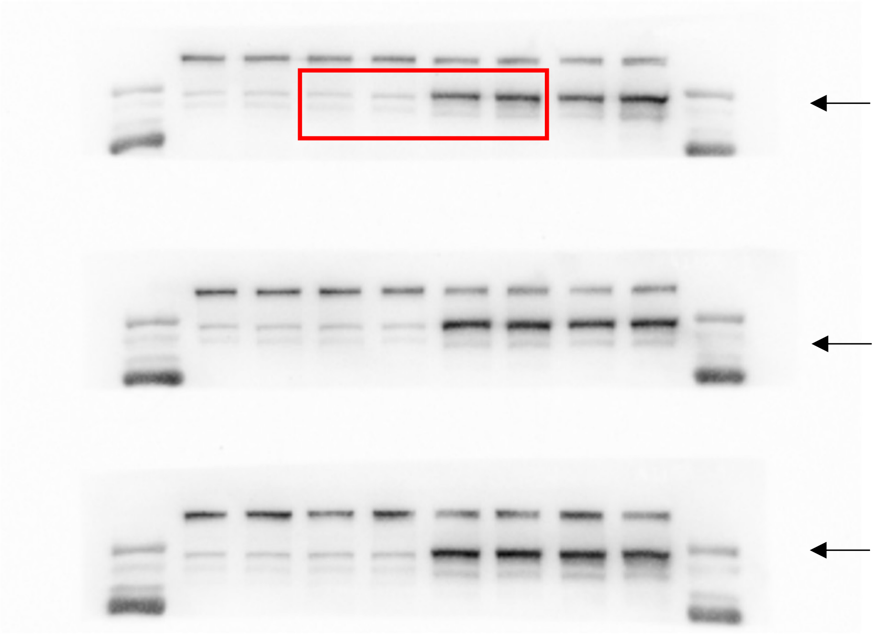

Membrane 1

Membrane 2

Membrane 3

HDAC5

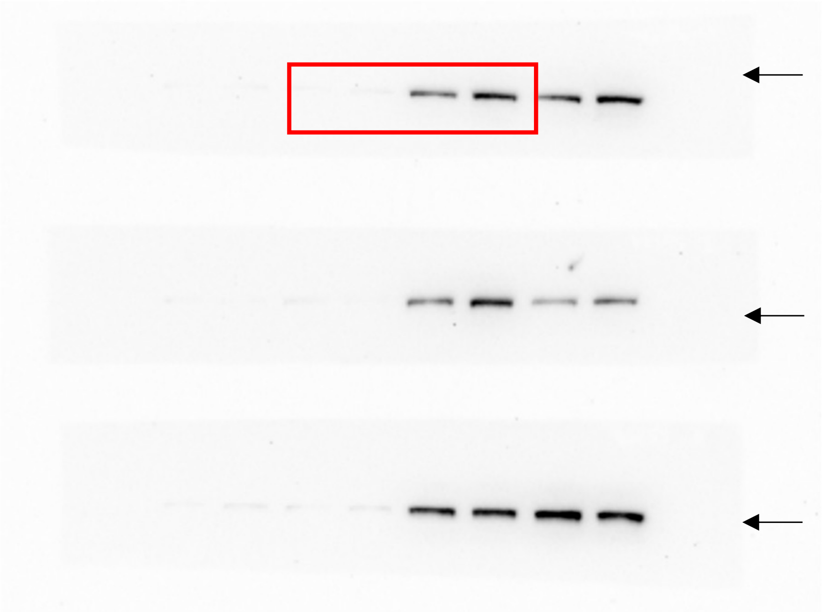

Membrane 1

Membrane 2

Membrane 3

Figure S3A

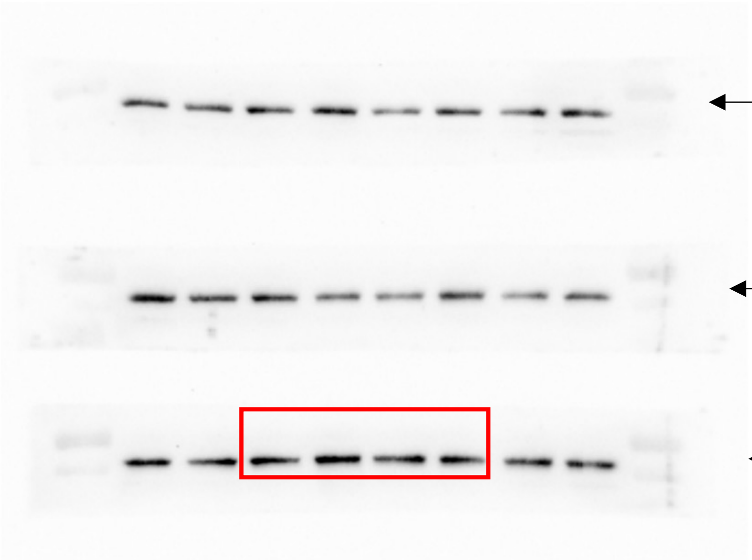

Membrane 3

Membrane 2

Membrane 1

Tubulin

Figure S3F

HDAC4

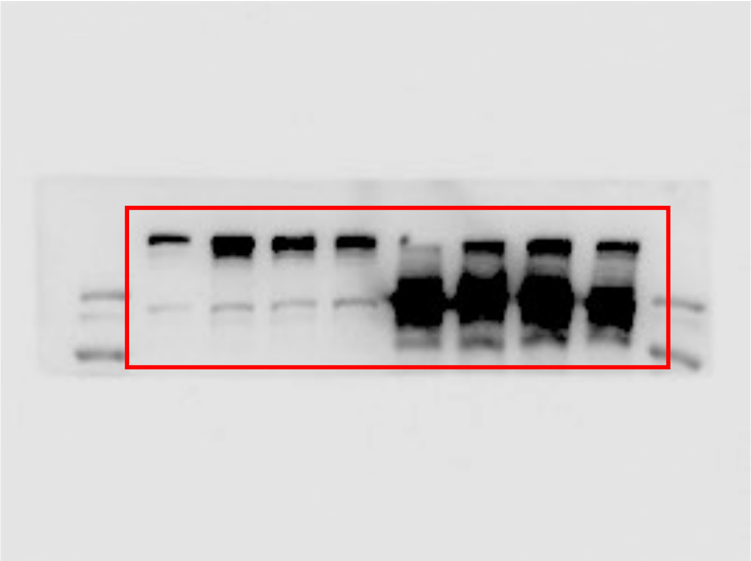

HDAC5

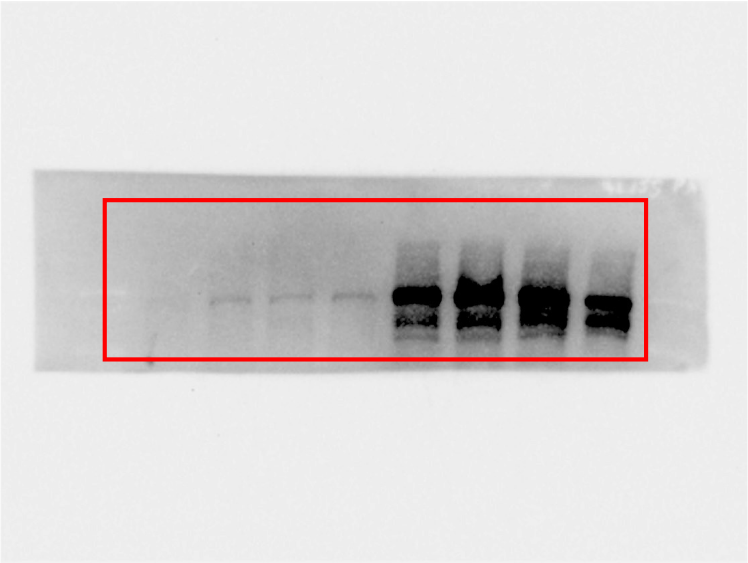

Supplement: Supplementary file 2 — Original Data File [file 41419_2023_6319_MOESM2_ESM.pdf]
